# Pterostilbene improves neurological dysfunction and neuroinflammation after ischaemic stroke via HDAC3/Nrf1-mediated microglial activation

**DOI:** 10.1186/s11658-024-00634-1

**Published:** 2024-08-28

**Authors:** Yuhua Chen, Wei He, Junlin Qiu, Yangyang Luo, Chenlong Jiang, Feng Zhao, Hong Wei, Jiao Meng, Tianlin Long, Xin Zhang, Lingjian Yang, Quanhua Xu, Juning Wang, Chi Zhang

**Affiliations:** 1 Department of Neurosurgery, Academy of Traditional Chinese Medicine, Bijie Traditional Chinese Medicine Hospital, Bijie, 551700 China; 2 Department of Medical Science Research Center, Peihua University, Xi’an, 710125 Shaanxi China; 3 Department of Neurosurgery, Qilu Hospital of Shandong University (Qingdao), Qingdao, 266000 Shandong China; 4 Department of Cardiology, First Hospital of Northwestern University, Xi’an, 710043 Shaanxi China; 5 School of Life Sciences, Northwest University, Xi’an, 710069 Shaanxi China; 6 Department of Sport Medicine, Sports Medicine Institute, Peking University Third Hospital, Beijing, 100191 China; 7 School of Chemistry & Chemical Engineering, Ankang University, Ankang, 725000 China; 8 Department of Neurosurgery, The Institute of Skull Base Surgery and Neurooncology at Hunan Province, Xiangya Hospital, Central South University, NO. 87 Xiangya Road, Changsha, 410008 China

**Keywords:** Ischaemic stroke, PTS, HDAC3, Nrf1 acetylation, Neuroinflammation

## Abstract

**Background:**

Stroke is a type of acute brain damage that can lead to a series of serious public health challenges. Demonstrating the molecular mechanism of stroke-related neural cell degeneration could help identify a more efficient treatment for stroke patients. Further elucidation of factors that regulate microglia and nuclear factor (erythroid-derived 2)-like 1 (Nrf1) may lead to a promising strategy for treating neuroinflammation after ischaemic stroke. In this study, we investigated the possible role of pterostilbene (PTS) in Nrf1 regulation in cell and animal models of ischaemia stroke.

**Methods:**

We administered PTS, ITSA1 (an HDAC activator) and RGFP966 (a selective HDAC3 inhibitor) in a mouse model of middle cerebral artery occlusion–reperfusion (MCAO/R) and a model of microglial oxygen‒glucose deprivation/reperfusion (OGD/R). The brain infarct size, neuroinflammation and microglial availability were also determined. Dual-luciferase reporter, Nrf1 protein stability and co-immunoprecipitation assays were conducted to analyse histone deacetylase 3 (HDAC3)/Nrf1-regulated Nrf1 in an OGD/R-induced microglial injury model.

**Results:**

We found that PTS decreased HDAC3 expression and activity, increased Nrf1 acetylation in the cell nucleus and inhibited the interaction of Nrf1 with p65 and p65 accumulation, which reduced infarct volume and neuroinflammation (iNOS/Arg1, TNF-α and IL-1β levels) after ischaemic stroke. Furthermore, the CSF1R inhibitor PLX5622 induced elimination of microglia and attenuated the therapeutic effect of PTS following MCAO/R. In the OGD/R model, PTS relieved OGD/R-induced microglial injury and TNF-α and IL-1β release, which were dependent on Nrf1 acetylation through the upregulation of HDAC3/Nrf1 signalling in microglia. However, the K105R or/and K139R mutants of Nrf1 counteracted the impact of PTS in the OGD/R-induced microglial injury model, which indicates that PTS treatment might be a promising strategy for ischaemia stroke therapy.

**Conclusion:**

The HDAC3/Nrf1 pathway regulates the stability and function of Nrf1 in microglial activation and neuroinflammation, which may depend on the acetylation of the lysine 105 and 139 residues in Nrf1. This mechanism was first identified as a potential regulatory mechanism of PTS-based neuroprotection in our research, which may provide new insight into further translational applications of natural products such as PTS.

**Supplementary Information:**

The online version contains supplementary material available at 10.1186/s11658-024-00634-1.

## Introduction

Stroke is considered a significant public health issue that profoundly impacts human well-being. Stroke remains a leading cause of death and permanent disability and thus imposes an annual global economic burden [1]. The number of stroke patients in China was the highest in 2019 [2]. Early diagnosis, timely intervention and treatment at specialized stroke centres markedly reduced global stroke-related incidence, recurrence and mortality from 2005 to 2019 [3, 4]. However, the incidence and mortality rates of stroke in China surpassed those of developed countries such as Britain, the USA and Japan during the same period [2, 5]. With an increasing population, challenges such as poor control of stroke-related risk factors persist, which increases the burden of stroke in China [5]. Compared with that in 2010, the incidence of stroke in China is projected to surge by approximately 50% by 2030 [5, 6]. Recombinant tissue plasminogen activator (tPA) remains the sole approved drug for the treatment of acute ischaemic stroke but has significant clinical limitations [1, 7]. Additionally, in recent years, researchers have increasingly focused on understanding the pathological mechanism and the repair of nerve function after stroke.

Generally, the neuroinflammatory response serves as a crucial secondary injury indicator in nerve injury and is linked with the activation and polarization of microglia [8, 9]. Targeting neuroinflammation holds promise for the treatment of brain injury [10–13]. As innate immune cells, microglia act as the “sentinels” of the central nervous system (CNS) [10]. Microglia are particularly sensitive to energy deficiency, as they swiftly transition from a quiescent state to an activated state upon brain stimulation by ischaemia [14, 15]. Activated microglia, which act as a double-edged sword in nerve recovery, can differentiate into M1- or M2-type microglia to maintain CNS homeostasis [16]. Classically activated cells of the M1 phenotype release various inflammatory cytokines and neurotoxic mediators (reactive oxygen and nitrogen species (ROS and RNS)), leading to blood‒brain barrier destruction, extracellular matrix degradation and exacerbation of cerebral ischaemic injury [16, 17]. Conversely, alternatively activated cells of the M2 phenotype secrete anti-inflammatory and nutritional factors, which decrease the local inflammatory response, protect nerve function and contribute to tissue preservation and nerve repair [16, 17]. Therefore, further understanding of the factors that regulate microglial polarization, inhibit M1-polarization of microglia and promote M2 polarization represents a promising strategy for treating neuroinflammation-related diseases, such as ischaemic stroke [18, 19].

Nuclear factor (erythroid-derived 2)-like 1 (Nrf1) and Nrf2 are two major transcription factors that govern intracellular redox homeostasis and are crucial for regulating resistance to both endogenous and exogenous stressors [20]. Nrf1, an indispensable redox determinant for maintaining mitochondrial homeostasis, orchestrates cellular redox equilibrium by integrating multiple regulatory networks via the MAPK, HIF1α, NF-κB, PI3K and AKT signalling pathways [21]. Nrf1-regulated microglial activation has been implicated in high-altitude cerebral oedema due to its ability to provoke NF-κB p65 and TFAM transcription [22]. Testosterone has been shown to mitigate the pulmonary epithelial inflammatory response in a rat model of chronic obstructive pulmonary disease by suppressing Nrf1-mediated NF-κB signalling and p65 phosphorylation [23]. Following traumatic brain injury (TBI), a decrease in Nrf1 acetylation levels and transcriptional activity leads to reduced mitochondrial mass in the pericontusional cortex [24]. The Nrf1/TFAM pathway plays a critical role in regulating mitochondrial biogenesis in response to subarachnoid haemorrhage, hypoxia–ischaemia and hyperoxia-induced brain injury [25–27]. Consequently, Nrf1 has emerged as a promising therapeutic target for stroke.

Pterostilbene (PTS), a derivative of resveratrol, is a phenolic compound initially extracted from sandalwood and subsequently discovered in fruits, such as blueberries and grapes, possesses anti-inflammatory and antioxidant properties [28, 29]. Our previous study revealed that PTS enhances neuronal protection against Aβ-induced neurotoxicity and cognitive dysfunction by modulating the PDE4A/CREB/BDNF pathway [30]. Previous research has revealed the neuroprotective effects of resveratrol in an ischaemic stroke model [31]. Resveratrol and PTS alleviate cardiovascular abnormalities in smokeless tobacco-induced oestrogen deficiency in female rats by increasing expression of the SIRT1, PGC-1α, PPAR-α, TFAM and Nrf-1 genes, increasing mtDNA transcription and by reducing cholesterol, LDH and TNF-α levels in cardiac tissue. However, PTS does not regulate SIRT1 levels [32]. Nonetheless, the mechanism by which PTS regulates Nrf1 signalling activation in microglia remains to be clarified. In this study, we aimed to explore the protective effect of PTS against neurological deficits and secondary injury against ischaemic stroke through histone deacetylase 3 (HDAC3)/Nrf1-mediated microglial actions, which might facilitate further translational studies.

## Materials and methods

### Animal experiment

All experimental procedures were approved by the Institutional Animal Care Committee of Xi’an Peihua University and were conducted in accordance with the Basel Declaration. Male C57BL/6 N mice (~8–10 weeks, weighing 22 ± 2 g) were maintained on a 12/12-h light/dark cycle (22 °C ± 2 °C and 40–70% humidity), with free access to food and water. All animals were randomized into the following groups by a computer (*n* = 18/group): sham, middle cerebral artery occlusion–reperfusion (MCAO/R) + vehicle, MCAO/R + PTS (1, 5, or 10 mg/kg), MCAO/R + ITSA1 (an HDAC activator), MCAO/R + RGFP966 (a selective HDAC3 inhibitor) and MCAO/R + PTS + ITSA1. Following the intraperitoneal injection of 50 mg/kg pentobarbital sodium, mice in every group except the sham group were subjected to MCAO, after which blood flow was re-established 2 h later. The body temperature of each mouse was maintained at 37 °C via thermostatic heating pads during and after the operation. In accordance with previous studies [33, 34], PTS, ITSA1 and RGFP966 were dissolved in 1% Tween 80 and administered via intraperitoneal injection immediately after reperfusion (PTS, 1 mg/kg (L), 5 mg/kg (M) or 10 mg/kg (H); ITSA1, 1 mg/kg; RGFP966, 10 mg/kg; vehicle, 1% Tween 80).

In another set of in vivo experiments, PLX5622 (1200 ppm) was part of an AIN-76A diet (MedChemExpress LLC, Shanghai, China), which was fed freely to deplete microglia; alternatively, AIN-76A chow was used as a control [35]. After 14 days, the mice were subjected to MCAO/R and intraperitoneally injected with PTS (10 mg/kg) immediately after reperfusion.

### Behavioural test

All animals underwent two neurological tests prior to surgery and at 24 and 72 h after surgery, and neurological and Garcia scores were determined via double-blind experiments according to methodologies described in our previous study [36]. The learning and memory functions of the animals were assessed using the Morris water maze at 7 days post-MCAO/R, as previously described. The circular pool was 120 cm in diameter and 50 cm deep, and the white platform was 6 cm in diameter and 30 cm in height. A water-based solution of titanium dioxide was injected into the pool approximately 1 cm above the platform. During the 5-day training period, the platform was placed in the third quadrant of the test site. All the mice were allowed 90 s to find the platform and 10 s to stay on it, or when no platform was found after 90 s, the mice were directed to the platform, where they could rest for 10 s. In the timed daily trial, animals were randomly placed in the first, second and fourth quadrants, with 4 min between tests. On day 6, each animal was tested for cognitive function from one direction. The average latency, swimming speed and swimming distance were recorded via SMART 3.0 software (Panlab, Barcelona, Spain).

### Inflammatory factor analysis

Brain tissue, primary microglia and culture medium supernatants were collected. After the protein concentration was measured with a bicinchoninic acid assay kit (Thermo Scientific, Waltham, MA, USA), a mouse TNF-α enzyme-linked immunosorbent assay (ELISA) Kit and a mouse IL-1β ELISA Kit (Beyotime Biotechnology, Shanghai, China) were used to measure TNF-α and IL-1β levels following the manufacturer’s instructions. Inducible nitric oxide synthase (iNOS) was examined with a mouse NOS2/iNOS ELISA Kit (Solarbio, Beijing, China).

### Histological analysis

The cerebral infarct volume was measured by 2,3,5-triphenyl tetrazolium chloride (TTC) staining. Fresh brain tissues were obtained at 24 h post-MCAO/R and maintained at −20 °C for 10 min, after which a series of 2-mm coronal slices were cut, stained with 2% TTC solution (Solarbio) at 37 °C for 30 min and fixed in 4% paraformaldehyde. The slices were photographed, and the infarct area was measured in a blinded manner with Image-Pro Plus (version 7.0).

### Immunofluorescence assays

After fixation, the slices were blocked with 5% BSA, incubated overnight with Nrf1 (Abcam, 1:100), HDAC3 (CST, 1:100), or IbA1 (CST, 1:1,000) primary antibodies and then incubated with secondary antibodies. The nuclei were stained with DAPI (Solarbio), and images were acquired via a fluorescence microscope (Leica, Oskar-Barnack, Germany).

### HDAC activity analysis

After the nuclear proteins were extracted from the cell lysates using a Nuclear Protein Extraction Kit (Solarbio), the HDAC enzyme activity in the 50 µg of lysates was analysed with an EpiQuik HDAC Activity/Inhibition Assay Kit (Colorimetric) (Epigentek, Farmingdale, NY, USA).

### Primary microglial culture

Microglia were obtained from mouse pups on postnatal day 1 according to a previous report [28]. Briefly, cerebral hemispheric tissues were minced and digested with 0.25% trypsin–EDTA (Thermo Scientific) for 25 min at 37 °C. In all, 5 × 10^5^ cells/ml were seeded into 24-well plates in DMEM (10% FBS) and were routinely cultured. On day 9, the plates of confluent mixed glial cultures were shaken at 180 rpm on an orbital shaker for 2 h at 37 °C. The medium containing purified microglia was collected, and the cells were resuspended in DMEM. They were then seeded into the wells of a 24-well plate and subjected to OGD/R treatment following procedures outlined in a previous report [33]. Following OGD/R, microglia were immediately treated with PTS (10 µM), ITSA1 (100 µM), or RGFP966 (10 µM) or left untreated for 24 h.

### Cell viability analysis

Microglial viability and death were assessed using CCK8 and LDH release assays. After treatment, 10 μl of CCK8 solution (Dojindo Laboratories, Tokyo, Japan) was added, the mixture was incubated at 37 °C for 4 h and the OD450 values were detected with a microplate reader (Thermo Scientific). The LDH Cytotoxicity Assay Kit (Beyotime Biotechnology) was used for the LDH release assay.

### Dual-luciferase reporter assay

The p65 promoter sequence was subsequently cloned and inserted into the pGl3 vector (Promega, Madison, WI, USA). HEK293T cells were transduced with Nrf1 [wild type (WT) or mutation], HDAC3 or overexpression vector and co-transfected with either the pGl3-p65 3′ untranslated region (UTR) luciferase reporter plasmid or the pRL-TK vector (Promega) expressing Renilla luciferase using Lipofectamine 3000 (Invitrogen). A total of 48 h after transfection, luciferase activity was determined via a Dual-Luciferase Reporter Assay System (Promega).

### Co-immunoprecipitation (co-IP)

The co-IP assay was performed using an Immunoprecipitation Kit with Protein A + G Magnetic Beads according to the manufacturer’s instructions (Beyotime Biotechnology, Shanghai, China). The samples were lysed in IP lysis buffer (Beyotime Biotechnology) containing a proteinase inhibitor cocktail (Beyotime Biotechnology). The lysate was subsequently centrifuged at 4 °C and 2500*g* for 5 min, after which the suspension was collected. An anti-Nrf1 (Abcam) antibody was coupled to the dynabeads and IgG was used as a negative control. Total protein was then mixed with the antibody-coupled dynabeads and incubated overnight at 4 °C. The dynabeads were adsorbed and washed, and the bound protein was eluted with 20 μl of eluent, mixed with 20 μl of 2× Laemmli buffer, and boiled for 5 min. Finally, western blotting was performed.

Endogenous or overexpressed Nrf1 protein degradation was assessed via a protein stability assay. Briefly, microglia treated with ITSA1 and untreated microglia were incubated with 100 μg/ml cycloheximide (CHX) (Sigma‒Aldrich) for 60 min. Similarly, microglia transfected for 48 h with Nrf1 mutants and microglia that were not transfected were also incubated with 100 μg/ml CHX for 60 min. The Nrf1 protein level was evaluated by western blot analysis. The western blot data were quantified via densitometry, and the levels were normalized to those of the control group without CHX treatment.

### Western blot analysis

The samples were lysed with lysis buffer (Beyotime), and nuclear cell lysates were extracted using the Nuclear Protein Extraction Kit (Solarbio). After protein concentration measurement, electrophoresis and transfer were performed, the membranes were blocked with 5% BSA and incubated overnight at 4 °C with the appropriate primary antibodies: HDAC1 (Proteintech, 1:1,000), HDAC2 (Proteintech, 1:1,000), HDAC3 (CST, 1:1,000), acetylated lysine (CST, 1:1,000), Nrf1 (Abcam, 1:1,000), iNOS (Proteintech, 1:1,000), Arg1 (Proteintech, 1:10,000), NF-κB p65 (CST, 1:1000), histone H3 (Proteintech, 1:10,000), FLAG (Proteintech, 1:10,000), β-actin (Proteintech, 1:10,000) and GAPDH (Proteintech, 1:10,000). Membranes were then incubated with horseradish peroxidase (HRP)-conjugated secondary antibodies (Abgent, 1: 20,000). The signal was revealed with an enhanced chemiluminescence (ECL) detection kit (Millipore, USA); full uncropped gel and blot images are shown in Figure S1.

### Quantitative real-time polymerase chain reaction (qRT–PCR) analysis

Total RNA was lysed by Trizol reagent (Invitrogen) and transcribed in reverse to complementary DNA by HiFi-MMLV cDNA first strand synthesis Kit (CW Bio, Beijing, China). The GoTaq qPCR Master Mix (Promega) was conducted to quantitative real-time PCR by CFX96TM Real-Time System (Bio-Rad). GAPDH was selected for internal control. The following primers were selected: HDAC1, TGATGCTGGGAGGAGGTG (forward: 5′-3′) and GTTGGAAGGGCTGATGTG (reverse: 5′-3′); HDAC2, TGACAAACC-AGAACACTCCAGAATA (forward: 5′-3′) and GAATAGCTTGCATTTGAACACCAG (reverse: 5′-3′); HDAC3, AGCCTTAATGCCTTCAACGTGG (forward: 5′-3′) and TCATTGACATAGCA-GAAGCCAGAGG (reverse: 5′-3′); Nrf1, TCTTGGAGTAAGTCGAGAAGTGT (forward: 5′-3′) and GTTGAAACTGAGCGAAAAAGGC (reverse: 5′-3′); and GAPDH, AAGACCCAGAAATGAAC (forward: 5′-3′) and TCTACACGATAACAACCA (reverse: 5′-3′).

### Statistical analysis

The results are expressed as the means ± standard deviations (SDs). All the statistical analyses were conducted with SPSS statistical software (version 21.0, IBM, Armonk, NY, USA). To compare differences between two groups, normally distributed continuous variables were compared using the student’s *t* test. For multiple comparisons of more than two groups, the data were analysed using one-way analysis of variance (ANOVA) followed by the Tukey‒Kramer post hoc test. *p* < 0.05 was considered significant.

## Results

### PTS improved motor behaviour, tissue infarction and neuroinflammation after ischaemia/reperfusion (I/R)

**Fig. 1:**
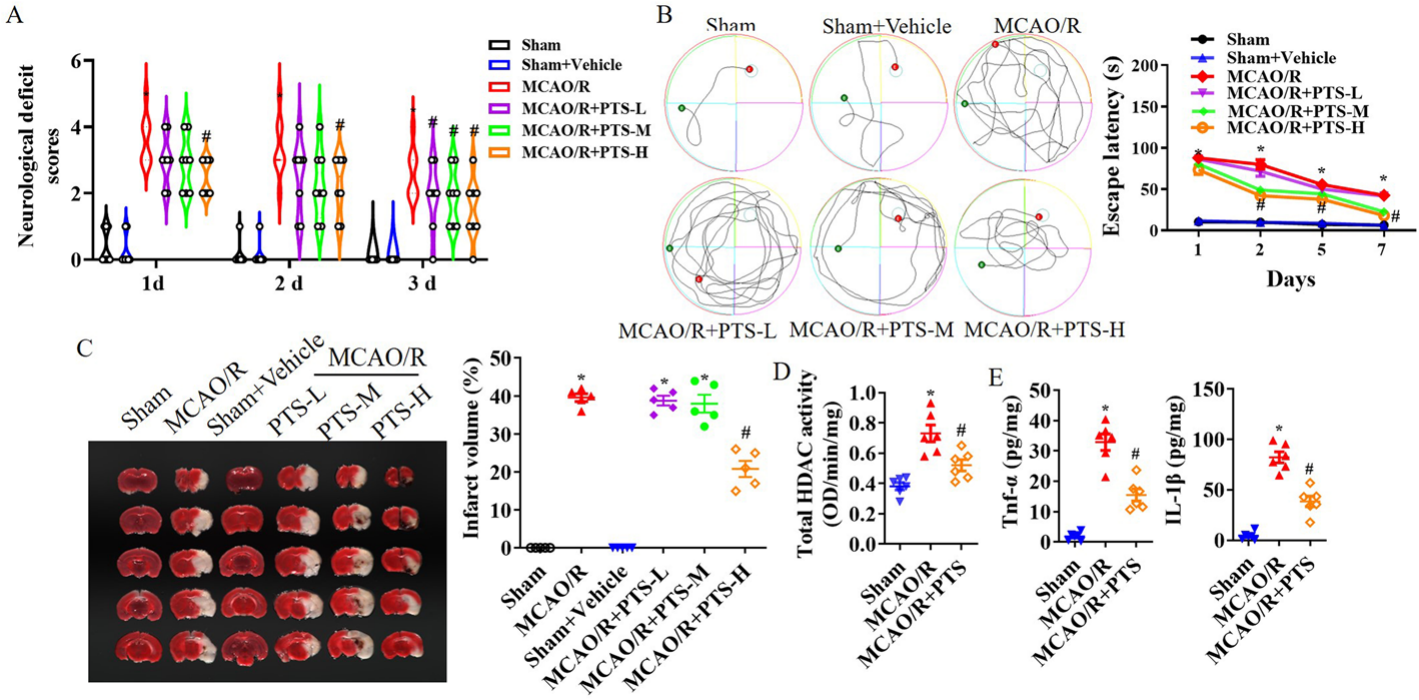
PTS improves motor behaviour and tissue infarction after I/R. PTS (1 mg/kg, 5 mg/kg or 10 mg/kg) was administered via intraperitoneal injection immediately after MCAO/R, and neurological tests were conducted twice before surgery and 1 day, 2 day and 3 day after MCAO/R, including assessment of the neurological deficit score (**A**) and performance in the hidden platform trial of the MWM (**B**). The data are presented as the means ± SEMs (*n* = 8). **C** Representative images of TTC staining at 24 h after MCAO/R and quantitative analysis of the hemispheric infarct ratio. Total HDAC activation (**D**) and the levels of the inflammatory factors TNF-α and IL-1β (**E**) in ischaemic brain tissue at 1 d after MCAO/R. The data are presented as the means ± standard error of the means (SEMs; *n* = 5). *p < 0.05, versus the sham group; #*p* < 0.05, versus the MCAO/R group. L, PTS 1 mg/kg; M, PTS 5 mg/kg; H, PTS 10 mg/kg

First, PTS was administered via intraperitoneal injection immediately after surgery, which was followed by assessment of neurological deficit and the hidden platform trial of the Morris water maze (MWM). Following MCAO/R, a significant neurological deficit was observed, and PTS administration improved neurological deficit scores in a concentration-dependent manner; notably, the best outcome was observed with 10 mg/kg PTS (*p* < 0.05; Fig. 1A). In the hidden platform trial of the MWM test conducted 1 day after MCAO/R, I/R led to an increase in the time taken by the mice to find the platform, whereas 10 mg/kg PTS resulted in a reduction in the latency time (*p* < 0.05; Fig. 1B). Additionally, TTC staining performed 1 day after MCAO/R revealed that the infarct size in the MCAO/R group was substantial; however, this effect was significantly attenuated in the 10 mg/kg PTS group (*p* < 0.05; Fig. 1C). Further analysis was conducted using 10 mg/kg PTS. High levels of HDAC activation and the inflammatory factors TNF-α and IL-1β were induced by I/R, but PTS administration mitigated these high levels following MCAO/R (see Fig. 1D and E).

### PTS facilitated Nrf1 expression by inhibiting HDAC3 after I/R

**Fig. 2:**
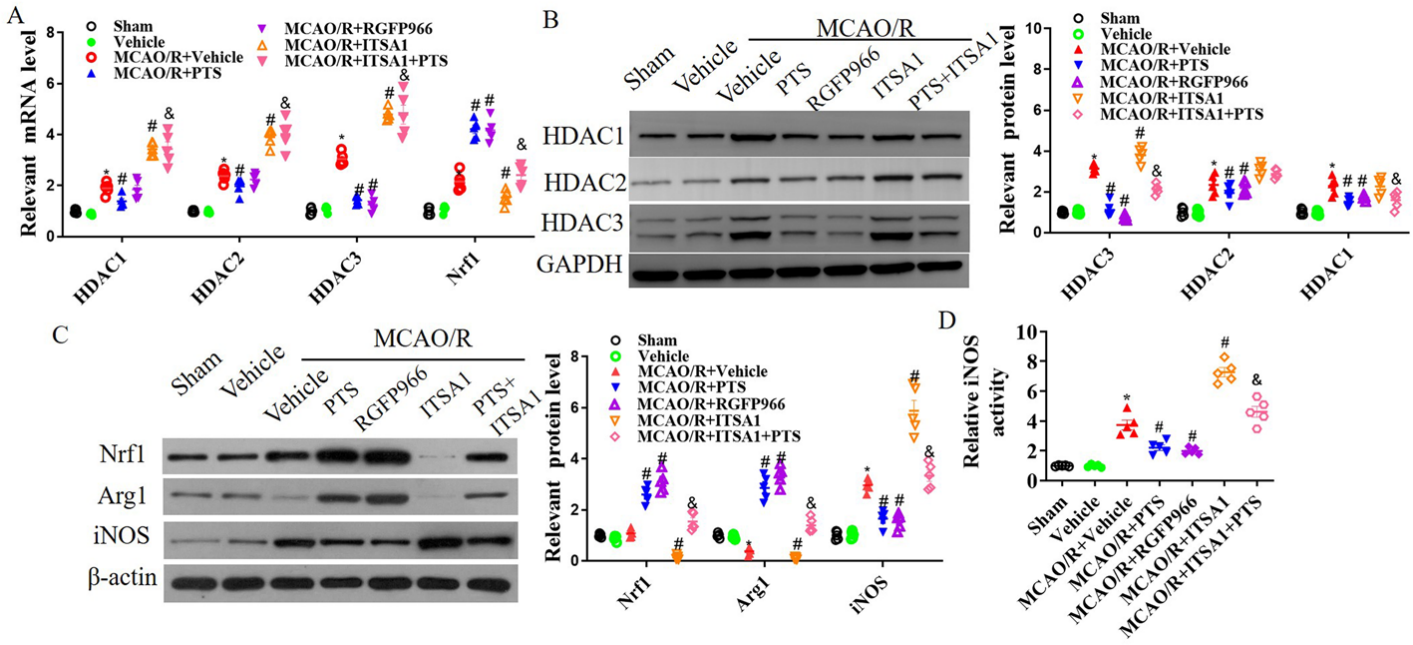
HDAC3 regulates Nrf1 expression and iNOS activation after I/R. After MCAO/R mice were treated with the HDAC activator ITSA1 and the HDAC3 inhibitor RGFP966, we conducted qRT‒PCR analysis of HDAC1, HDAC2, HDAC3 and Nrf1 mRNA levels (**A**); WB analysis of HDAC1, HDAC2 and HDAC3 expression (**B**); WB analysis of Nrf1, iNOS and Arg1 expression (**C**); and ELISA analysis of iNOS activation (**D**) in total cell lysates of ischaemic brain tissue 24 h after MCAO/R. The data are presented as the means ± SEMs (*n* = 5). *p < 0.05, versus the sham group; #*p* < 0.05, versus the MCAO + vehicle group; &*p* < 0.05, versus the MCAO/R + PTS group

**Fig. 3:**
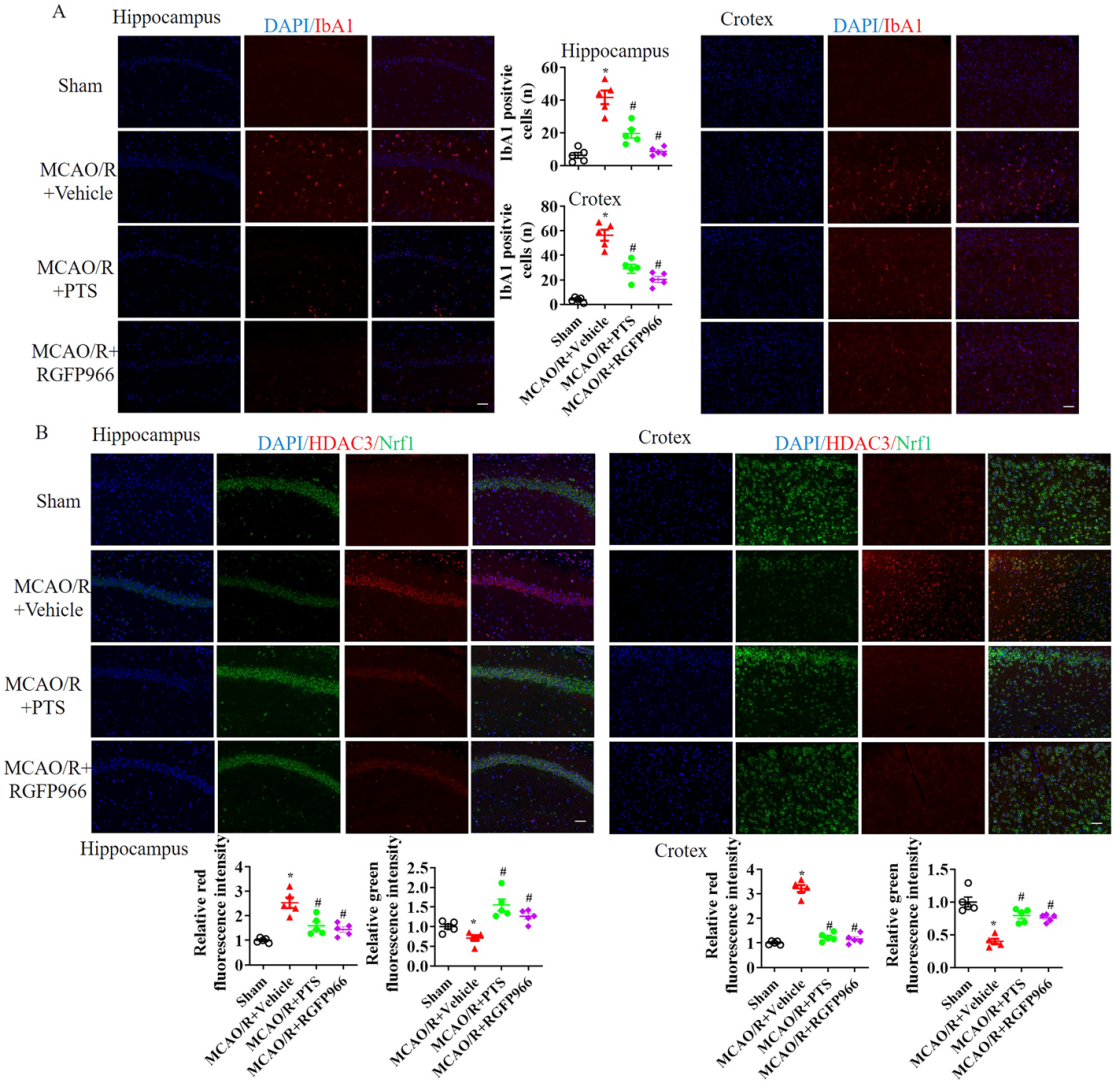
PTS induces Nrf1 and reduces IbA1 after I/R. At 24 h after MCAO/R, IF analysis of IbA1 expression (**A**) and HDAC3 and Nrf1 expression (**B**) in the hippocampus and cortex. Nuclei were stained with DAPI, *n* = 5; scale bar: 20 μm

Due to the alteration in HDAC activity following PTS treatment, we further treated MCAO/R mice with the HDAC activator ITSA1 and the HDAC3 inhibitor RGFP966. The mRNA levels of HDAC1, HDAC2, HDAC3A and Nrf1 were evaluated in ischaemic brain tissue (Fig. 2A), which revealed upregulation at 24 h post-MCAO/R. Notably, HDAC3 was more strongly upregulated, was significantly inhibited by PTS and RGFP966 treatment, and its upregulated expression was reversed by ITSA1 treatment. Similarly, Nrf1 mRNA levels were stimulated by PTS and RGFP966 but extinguished by ITSA1 following MCOA/R (see Fig. 2A). After MCAO/R, PTS and RGFP966 more strongly inhibited HDAC3 expression than HDAC1 expression, whereas ITSA1 increased HDAC3 expression, which limited the beneficial effects of PTS on HDAC3 (Fig. 2B). Subsequently, Nrf1, iNOS and Arg1 expression was measured. PTS and RGFP966 increased Nrf1 and Arg1 expression and decreased iNOS expression and activation post-MCAO/R. Conversely, ITSA1 exerted opposing effects and hindered the beneficial impact of PTS on Nrf1 and Arg1 upregulation and iNOS inhibition (Fig. 2C and D). Furthermore, HDAC3, Nrf1 and IbA1 expression was analysed via immunofluorescence analysis 24 h after I/R injury. I/R induced HDAC3 expression while dampening Nrf1 expression in the hippocampus and cortex. However, PTS and RGFP9669 suppressed HDAC3 expression and enhanced Nrf1 expression after MCAO/R (Fig. 3A). Additionally, IbA1 was upregulated in the MCAO/R group, but this upregulation was alleviated in the MCAO/R + PTS and MCAO/R + RGFP9669 groups (Fig. 3B).

**Fig. 4:**
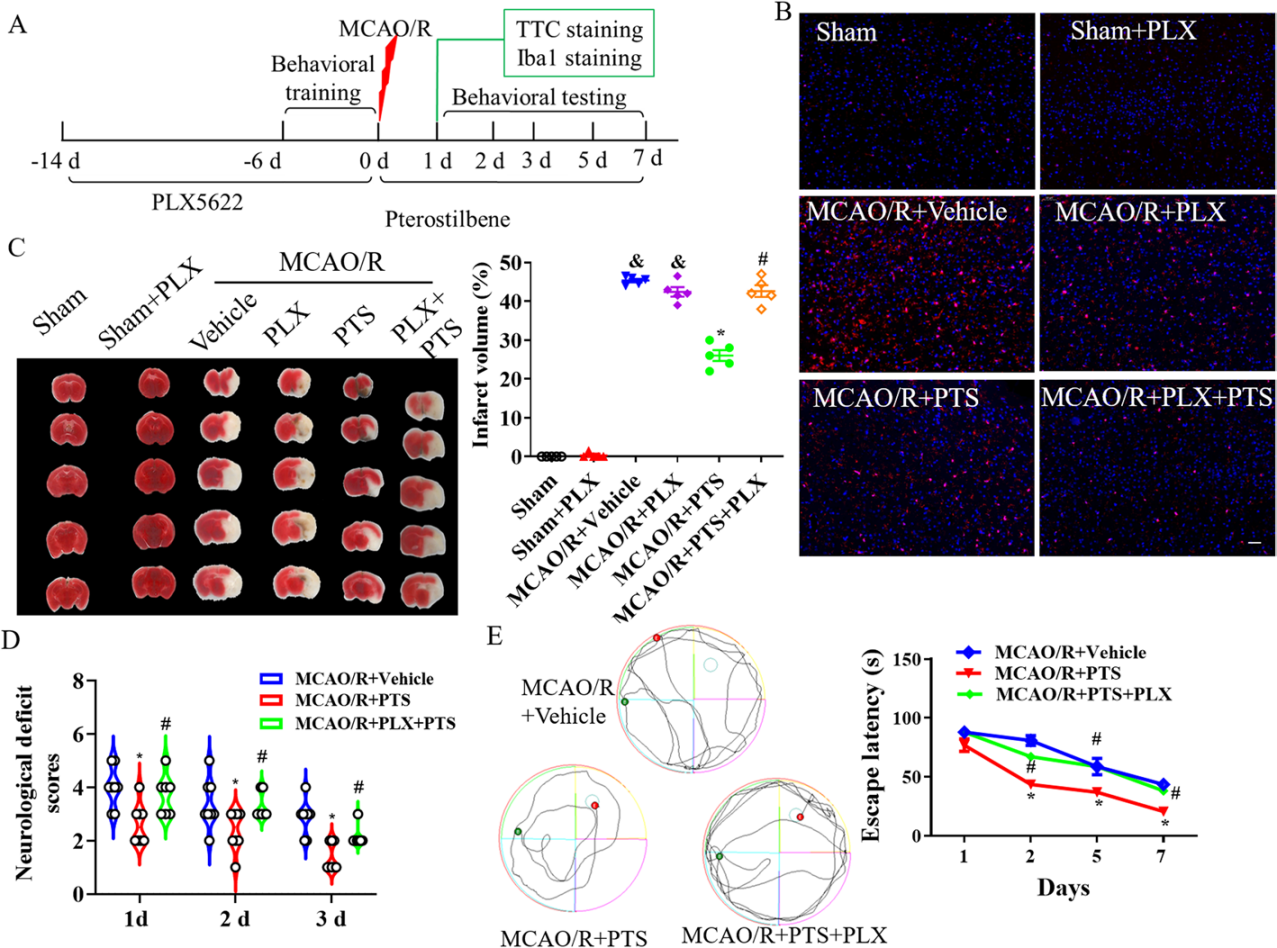
Microglia elimination with the CSF1R antagonist PLX5622 attenuated the therapeutic effects of PTS following I/R in vivo. **A** Overview of the timeline of in vivo experiments. Before MCAO/R, the mice were fed a PLX5622 (PLX) AIN-76A diet or AIN-76A chow for 14 days. **B** IF analysis of Iba1 staining in the brain after MCAO/R; the cell nuclei are shown in blue (DAPI). Scale bar = 20 μm, *n* = 5. **C** Representative images of TTC staining at 24 h after MCAO/R and quantitative analysis of the hemispheric infarct ratio (*n* = 5). Neurological tests, including assessment of the neurological deficit score (**D**) and performance in the hidden platform trial of the MWM (**E**), were conducted twice before surgery and at 1, 2 and 3 days after MCAO/R (*n* = 8). The data are presented as the means ± SEMs (*n* = 5). *p* < 0.05, versus the sham group; *p < 0.05, versus the MCAO + vehicle group; #*p* < 0.05, versus the MCAO/R + PTS group

To explore the effects of microglial elimination on the therapeutic effect of PTS following MCAO/R, the CSF1R inhibitor PLX5622 was used to deplete microglia, the timeline of which is presented in Fig. 4A. Dietary administration of PLX5622 beginning 14 days before MCAO/R resulted in the infrequent observation of Iba1 + microglia (Fig. 4B). I/R activated microglia in the MCAO/R + vehicle group, while PLX5622 and PTS reversed the activation of microglia (Fig. 4B). Microglial depletion by PLX5622 resulted in infarct sizes that were comparable between the MCAO/R + vehicle group and the other groups (Fig. 4C). Tissue infarct size decreased with PTS treatment at 24 h after MCAO/R, but the elimination of microglia counteracted the protective effect of PTS in the MCAO/R + PLX5622 + PTS group (*p* < 0.05, Fig. 4C). According to previous studies [34], the administration of PLX5622 did not affect animal behaviour, and we also observed no differences between the sham group and the sham + PLX5622 group. After MCAO/R, PLX5622 administration partly attenuated the efficacy of PTS treatment and exacerbated the neurobehavioural deficit score (*p* < 0.05, Fig. 4D) and escape latency (*p* < 0.05, Fig. 4E). These findings suggest that the therapeutic effect of PTS in I/R may be related to microglial activation.

**Fig. 5:**
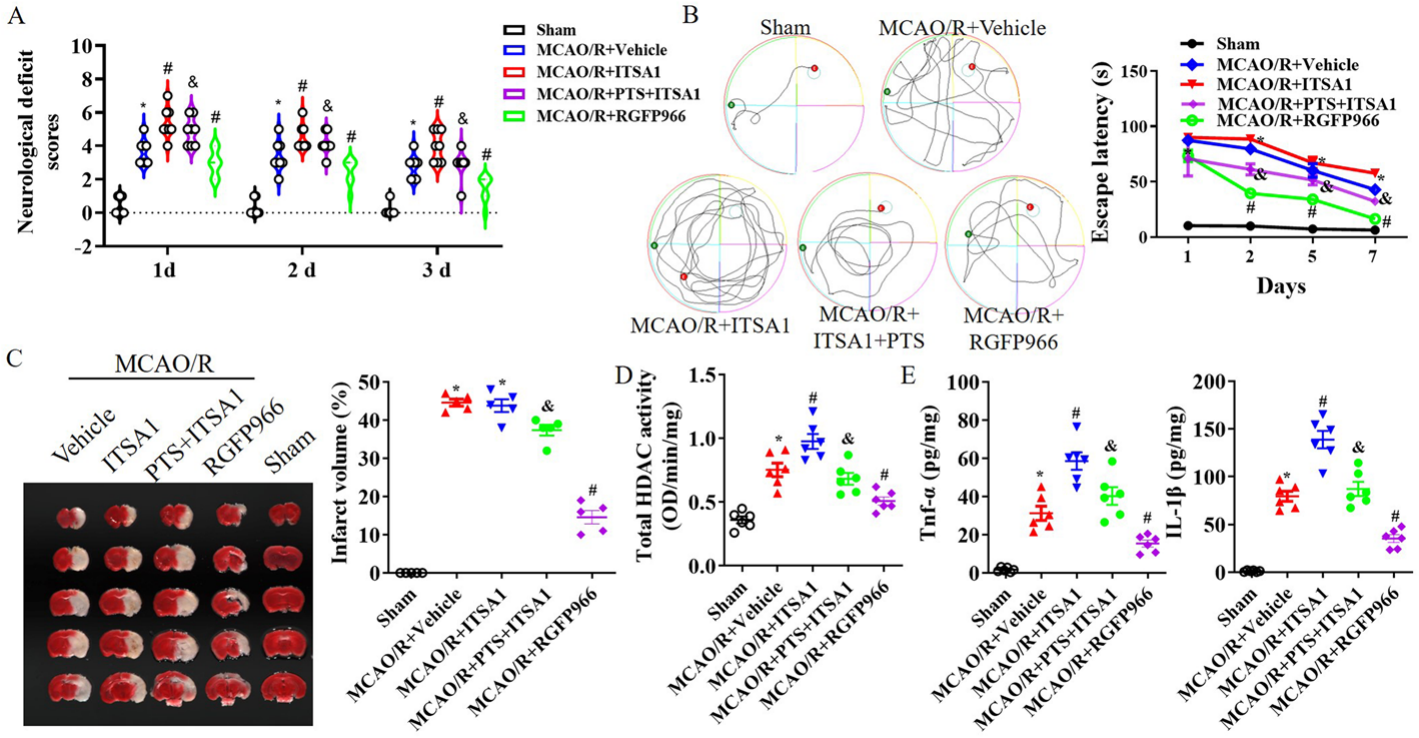
HDAC3 inhibition improves motor behaviour and tissue infarction after I/R. After PTS, ITSA1 and/or RGFP966 were administered via intraperitoneal injection, MCAO/R mice underwent neurological tests twice before surgery and at 1, 2 and 3 days after MCAO/R; testing included assessment of the neurological deficit score (**A**) and performance in the hidden platform trial of the MWM (**B**). The data are presented as the means ± SEMs (*n* = 8). **C** Representative images of TTC staining at 24 h after MCAO/R and quantitative analysis of the hemispheric infarct ratio. HDAC activation (**D**) and the levels of the inflammatory factors TNF-α and IL-1β (**E**) in ischaemic brain tissue 1 day after MCAO/R. The data are presented as the means ± SEMs (*n* = 5). *p < 0.05, versus the sham group; #*p* < 0.05, versus the MCAO/R + vehicle group; &*p* < 0.05, versus the MCAO/R + ITSA1 group

In MCAO/R mice, we also assessed motor behaviour, tissue infarction and neuroinflammation following HDAC3 regulation. RGFP9669 improved neurological deficits, motor behaviour and infarct size, while HDAC activation by ITSA1 reversed the neuroprotective effects of PTS after MCAO/R (*p* < 0.05, Fig. 5A–C). RGFP9669 reduced HDAC activation and the levels of the inflammatory factors TNF-α and IL-1β following I/R injury; these were increased by ITSA1, which reversed the PTS-mediated decreases in HDAC activation and TNF-α and IL-1β levels after MCAO/R (*p* < 0.05, Fig. 5D and E).

Therefore, these data suggest that the improvements in Nrf1 expression and neurological function caused by PTS may be attributed to HDAC3 inhibition in cerebral I/R injury.

### HDAC3-mediated Nrf1 deacetylation is involved in the ability of PTS to improve neuroinflammation

**Fig. 6:**
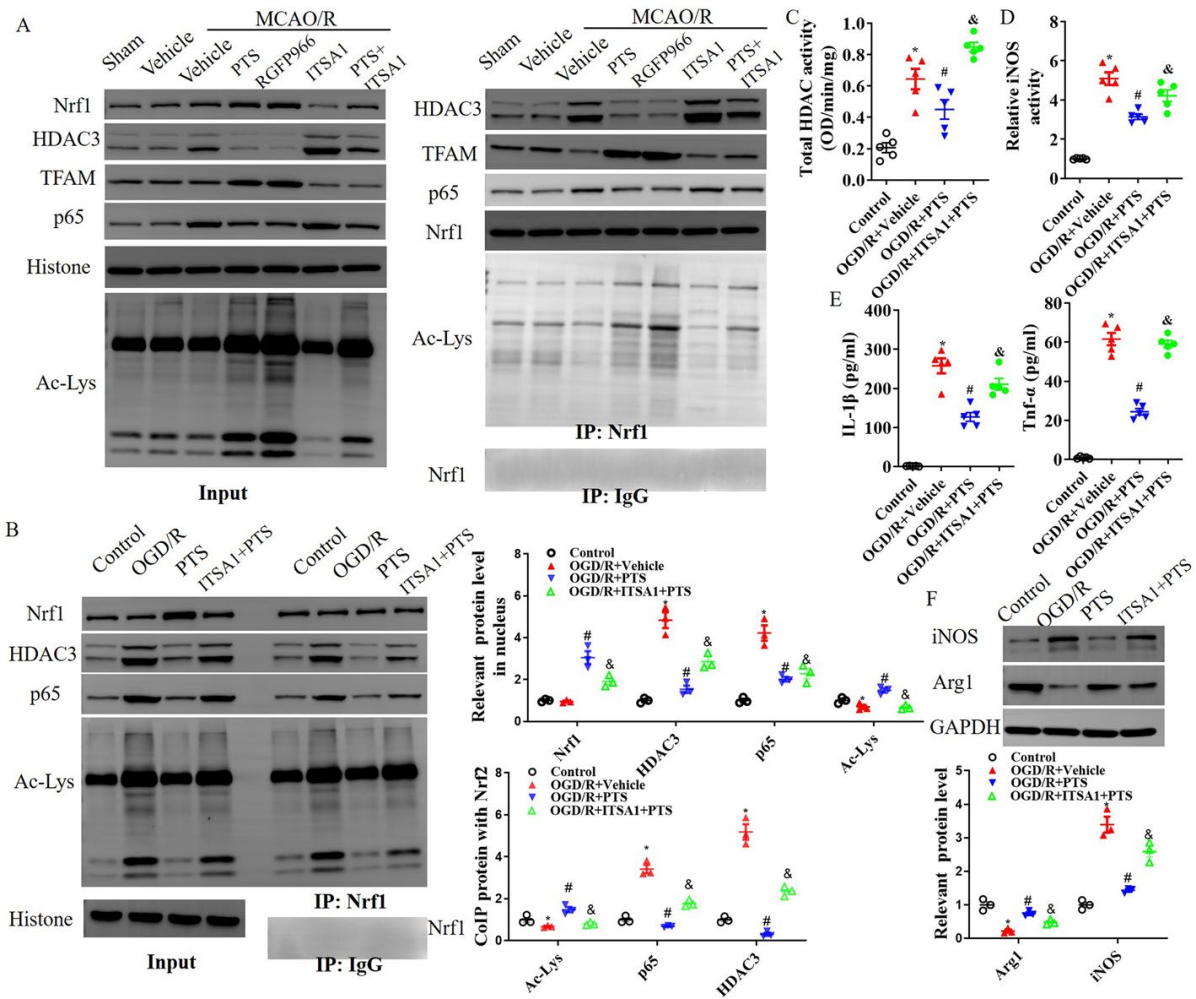
PTS improves Nrf1 acetylation and microglial activation by inhibiting HDAC3. **A** Nrf1 acetylation in the cell nucleus of ischaemic brain tissue was detected by a co-IP assay 24 h after MCAO/R. The data are presented as the means ± SEMs (*n* = 5). *p < 0.05, versus the sham group; #*p* < 0.05, versus the MCAO/R + vehicle group; *p* < 0.05, versus the MCAO/R + ITSA1 group. After OGD/R, microglia were immediately treated with PTS, ITSA1 + PTS, or vehicle for 24 h in vitro. Then, the acetylation of Nrf1 in the cell nuclei of microglia was detected by co-IP (**B**). Total HDAC activation (**C**), iNOS activation (**D**) and the levels of the inflammatory factors TNF-α and IL-1β were determined by ELISA (**E**), and WB analysis of iNOS and Arg1 expression (**F**) was performed using total cell lysates from OGD/R-induced microglia. The data are presented as the means ± SEMs (*n* = 3). *p < 0.05, versus the control group; #*p* < 0.05, versus the OGD/R + vehicle group; *p* < 0.05, versus the OGD/R + PTS group

To further elucidate the role of PTS in Nrf1 acetylation, we assessed Nrf1 and HDAC3 expression in the nucleus and investigated the interaction between Nrf1 and HDAC3 and acetylation using co-IP. PTS and RGFP966 reduced HDAC3 expression and increased lysine acetylation in the nucleus, which reversed I/R-induced binding of HDAC3 and Nrf1 and Nrf1 acetylation. Conversely, ITSA1 exacerbated the binding of HDAC3 and Nrf1 and reduced Nrf1 acetylation after MCAO/R, which impaired the beneficial effect of PTS on Nrf1 acetylation (Fig. 6A). We also detected Nrf1 acetylation in the nuclei of microglia after OGD/R. OGD/R induced HDAC3 and p65 expression and inhibited lysine acetylation in the nucleus, but PTS reversed the OGD/R-induced binding of HDAC3 and Nrf1 and Nrf1 acetylation. Conversely, ITSA1 inhibited the ability of PTS to improve the OGD/R-induced increase in Nrf1 acetylation (Fig. 6B). In addition, PTS suppressed the OGD/R-induced iNOS expression and activation, total HDAC activation, and the levels of the inflammatory factors TNF-α and IL-1β, but these effects were blocked by ITSA1 treatment (Fig. 6C–F).

**Fig. 7:**
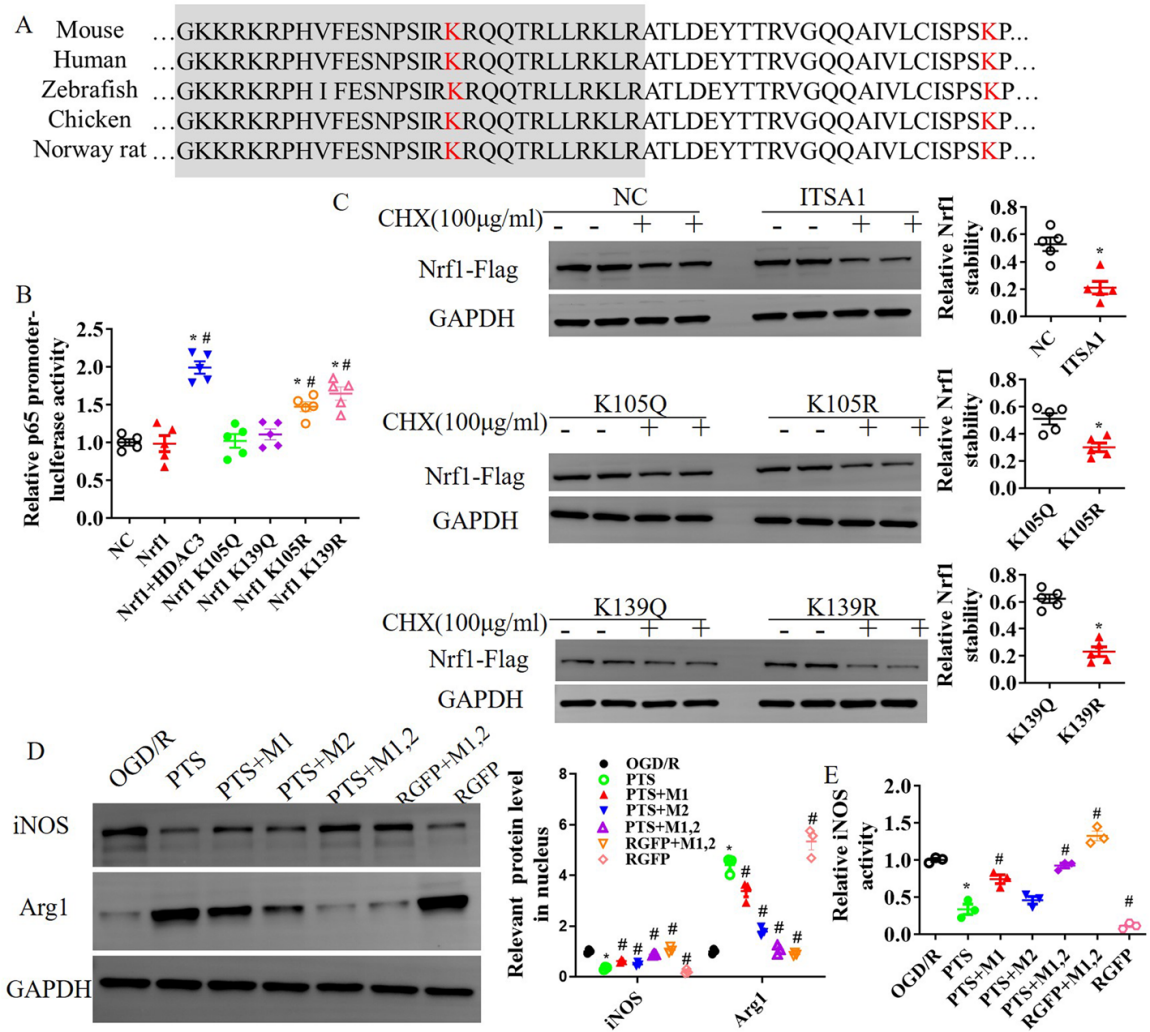
Nrf1 acetylation affects Nrf1 stability and microglial activation. **A** Lysine mutation design of Nrf1. **B** Luciferase activity was measured in microglia transfected with a murine p65 promoter–luciferase reporter constructed along with WT Nrf1 or Nrf1 mutants. HDAC3 was co-expressed as indicated. The p65 promoter reporter activity was normalized to Renilla luciferase activity and empty vector (NC) activity. Luciferase activity was measured and analysed. An empty vector was used as a control, and pairwise comparisons were made between the glutamate and arginine mutants for each amino acid site (*n* = 5 per group). **C** Protein stability in microglia transfected with the K105Q, K105R, K139Q and K139R mutants after treatment with 100 μg/ml CHX. Samples were obtained 0 and 90 min after CHX treatment (*n* = 3 per group). In microglia with OGD/R-induced injury, the influence of the Nrf1 K105R and/or K139R mutations on the improvement observed after PTS treatment were evaluated by WB analysis of iNOS and Arg1 (**D**) and ELISA analysis of iNOS activation (**E**). The data are presented as the means ± SEMs (*n* = 3). *p < 0.05, versus the control group; #*p* < 0.05, versus the OGD/R + vehicle group; *p* < 0.05, versus the OGD/R + PTS group

**Fig. 8:**
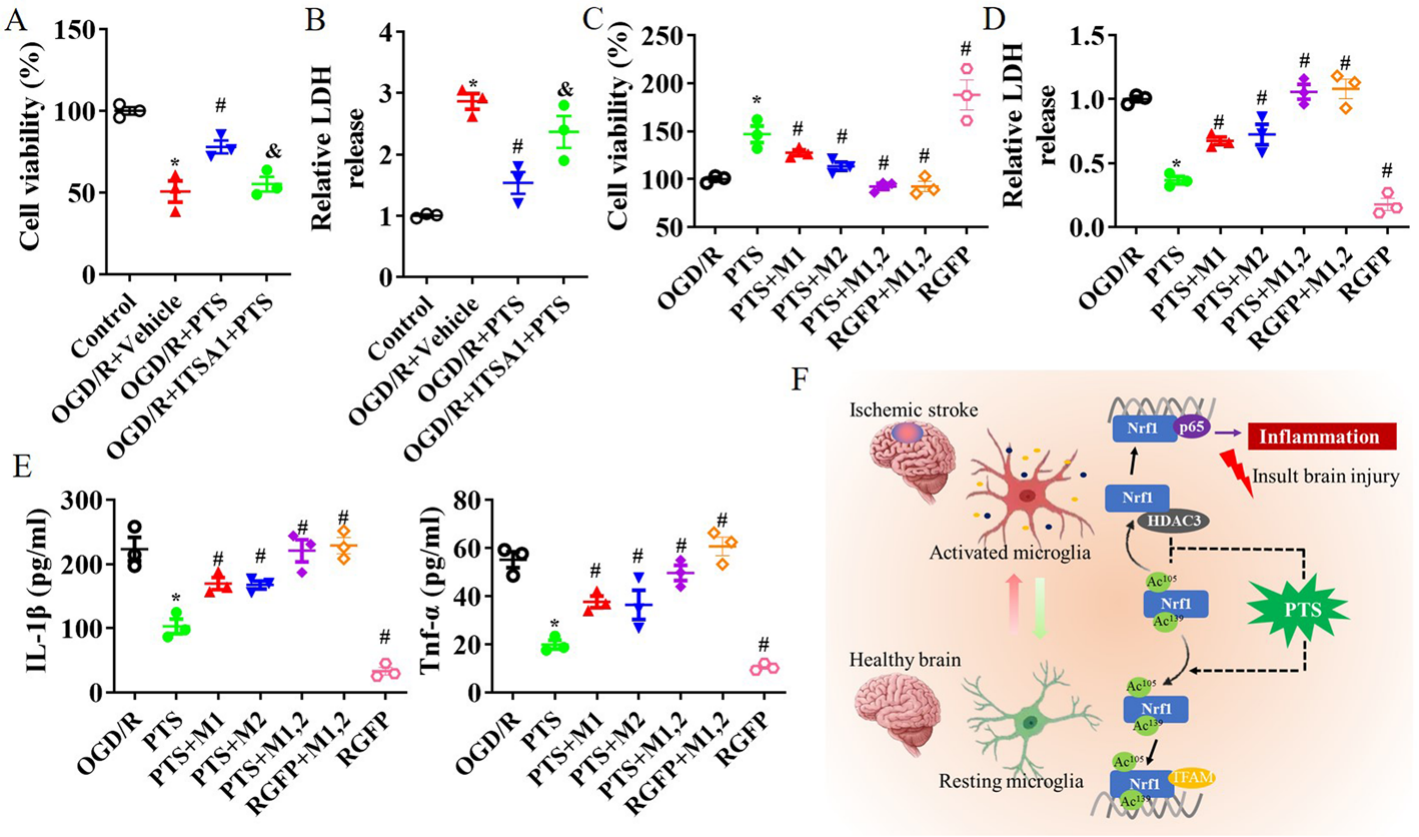
Nrf1 acetylation mediates microglial injury and TNF-α and IL-1β release. After treatment with PTS or ITSA1, a CCK8 assay (**A**) and an LDH release assay (**B**) were used to assess microglial injury in the OGD/R-induced microglial injury model; *p < 0.05, versus the control group; #*p* < 0.05, versus the OGD/R + vehicle group; *p* < 0.05, versus the OGD/R + PTS group. After treatment with PTS or RGFP966, a CCK8 assay (**C**) and an LDH release assay were performed (**D**), and the levels of the inflammatory factors TNF-α and IL-1β (**E**) were detected in OGD/R-induced microglia with the Nrf1 K105R and/or K139R mutation. **F** Model of microglial inflammation induced by PTS-mediated HDACA/Nrf1 after ischaemic stroke. *p < 0.05, versus the OGD/R group; #*p* < 0.05, versus the PTS group. The data are presented as the means ± SEMs (*n* = 3)

To investigate the specific lysine residues of Nrf1 that are regulated by HDAC3, we mutated two lysine residues to glutamine or arginine and then measured activation of the p65 gene promoter and the stability of the Nrf1 protein. Nrf1 contains a conserved lysine-rich sequence, and the “GKKRKRPHVFESNPSIRKRQQTRLLRKLR” sequence of mouse Nrf1 is a nuclear localization signal that is essential for proper protein function. As depicted in Fig. 7A, the lysine mutations in mouse Nrf1 were at K105 and K139. We observed that the K105 and K139 mutations affected p65 gene promoter activity. Specifically, the K105Q or K139Q mutation resulted in similar transcriptional activity to that in the Nrf1 WT group, but the K105R or K139R mutation resulted in increased transcriptional activation, and compared with WT Nrf1 alone, the co-expression of HDAC3 with WT Nrf1 also significantly increased p65 gene promoter activity (Fig. 7B). Furthermore, HDAC3 increased the instability of Nrf1 in response to CHX, and consistent with the luciferase assay data, the K105R and K139R mutants also decreased the stability of the Nrf1 protein in response to CHX (Fig. 7C). Using protein and factor analysis, mutation of K105R and/or K139R reversed the ability of PTS to alleviate OGD/R-induced iNOS expression and activation. Moreover, the combination of the K105R and K139R mutations nullified the improvement in microglial activation provided by the HDAC3 inhibitor RGFP966 in vitro (see Fig. 7D and E). Furthermore, PTS was found to ameliorate OGD/R-induced microglial injury, but this effect was inhibited by ITSA1 treatment (see Fig. 8A and B). The protective effects of PTS on microglial injury and the release of the inflammatory factors TNF-α and IL-1β depended on Nrf1 acetylation. However, the K105R and/or K139R mutation counteracted this positive effect in the OGD/R-induced microglial injury model. Additionally, the combination of the K105R and K139R mutations negated the improvements in microglial injury and neuroinflammation induced by the HDAC3 inhibitor RGFP966 (Fig. 8C–E). Thus, the results suggest that lysines 105 and 139 of Nrf1 may be functional deacetylation targets of HDAC3, which regulates the stability and function of Nrf1 in microglial activation and neuroinflammation.

## Discussion

Cerebral I/R leads to cell death, neuronal overexcitation, oxidative stress and neuroinflammation, which are key factors that lead to cerebral I/R injury and secondary injury [3, 36, 37]. In this study, we demonstrated that PTS attenuated ischaemia-induced brain infarction, neuroinflammation and neurological deficits in an MCAO/R mouse model. In an in vitro OGD/R model, PTS protected against OGD/R-induced microglial injury and the production of proinflammatory molecules, as seen in ischaemic conditions, through the upregulation of HDAC3/Nrf1 signalling in microglia (Fig. 8F), which suggests that PTS is a promising strategy for ischaemia stroke.

Recently, the neuroprotective effects of PTS in stroke have been demonstrated and are attributed to its anti-inflammatory action [33, 38]. We observed that PTS protected against neuronal damage and suppressed ischaemic stroke-induced changes in the levels of TNF-α, IL-1β and iNOS. OGD/R-induced changes in the levels of iNOS/Arg1 and inflammatory responses by microglia, and the therapeutic effect of PTS in I/R was counteracted after microglial depletion with PLX5622 treatment. CSF1R is a critical regulator of immunosuppressive macrophage expansion [39], and studies using the CSF1R inhibitors PLX3397 and PLX5622 have revealed an important role of microglial consumption in inflammation regulation and nerve damage [40, 41]. ACT001 attenuates microglia-mediated neuroinflammation after traumatic brain injury [35], and CD22 blockade modulates microglial activity to suppress neuroinflammation following intracerebral haemorrhage [42]. Therefore, the effects of PTS may be related to the promotion of M2 polarization in microglia. Neuroinflammatory responses exacerbate brain damage following ischaemic stroke and are driven by the release of reactive oxygen species (ROS), chemokines and cytokines [43]. Under physiological conditions, a delicate balance exists between proinflammatory and anti-inflammatory cytokines; however, this balance is disrupted in the early stages of acute ischaemic stroke [44]. Within 6 to 12 h after cerebral ischaemia, the levels of TNF-α, IL-1β and IL-6 are increased in stroke patients and are associated with more severe neurological symptoms and poorer outcomes [45, 46]. Ischaemic stroke triggers the transformation of microglia into the M1 phenotype, after which they secrete IL-1β and exert neurotoxic effects. Conversely, TGF-β and IL-10 polarize microglia into the M2 type to mitigate potential damage through the production of various mediators [47, 48].

According to previous studies, ischaemia/reoxygenation induces excessive accumulation of ROS, electrophilic molecules and protein toxic stress; this activates Nrf1 and the specific Nrf1 downstream protein TFAM, which counteracts mitochondrial function and cell death [25, 26, 49]. Both endogenous and exogenous induction of Nrf1 activation have been shown to effectively protect against nerve injury [25, 26]. Additionally, electroacupuncture therapy has been found to successfully improve depression-like behaviour and cognitive dysfunction while inducing the Nrf1/TFAM pathway after I/R injury [50]. In mice, the specific knockdown of Nrf1 in brown adipocytes results in endoplasmic reticulum stress, tissue inflammation and markedly diminished mitochondrial function [51]. Furthermore, Nrf1 downregulation in RAW264.7 cells results in increased M1 polarization (including increases in IL-6, IL-1β, COX-2 and CCL2 levels) through disinhibition of STAT1/3 but not via the NF-κB, ERK1/2 and/or p38 signalling pathways [52]. Nrf-1/TFAM is positively correlated with increases in the levels of microglial M2 phenotype markers, including Arg1 and brain-derived neurotrophic factor (BDNF), and is negatively correlated with decreases in the levels of M1 phenotype markers, including iNOS and TNF-α, after LPS + IFNγ treatment of N9 microglia [53]. We found that PTS decreased NF-κB p56 accumulation and the interaction of Nrf1 and NF-κB p65 but increased the expression of Nrf1 and TFAM in the cell nucleus, as well as Arg1/iNOS expression in brain tissue, after ischaemia. In OGD/R-induced microglia, PTS also suppressed the interaction of Nrf1 with NF-κB p65 and NF-κB p56 accumulation in the cell nucleus, which meditated microglial activation and the levels of inflammatory factors. PTS decreases infarct volume, brain oedema and neuronal death and improves long-term neurological function by inhibiting the phosphorylation and nuclear translocation of NF-κB-mediated oxidation and inflammatory mediators [38]. Liu et al. demonstrated that PTS improves cerebral I/R injury in rats via inhibition of the ROS/NF-κB-regulated inflammatory response of microglia [33]. Pradeepkiran et al. reported that resveratrol decreases the inflammatory response and oxidative stress and increases mitochondrial biogenesis via the upregulation of SIRT1 and SIRT3 and the activation of Nrf1, which suppresses NF-κB signalling [54]. Accordingly, we believe that Nrf1/TFAM-mediated NF-κB signalling is involved in the promoting role of PTS in neuroinflammation and M2 polarization of microglia.

Previous studies reported that the Nrf1 signalling pathway cooperates with NF-κB signalling and participates in inflammation regulation [22, 23]. Hypoxia overactivates microglia via Nrf1, which activates the transcription of NF-κB p65 and TFAM [22], and Nrf1 is involved in LPS-mediated acute lung injury and inflammatory responses, including regulation of L-1β and IL-6 levels, via the transcriptional regulation of NF-κB p65 [55]. NF-κB binding to intron 1 enhances Nrf1 promoter activity [56]. LMP2A-regulated Nrf1 expression maintains latent infection by Epstein–Barr virus through the NF-κB pathway [57]. Therefore, the regulatory role of Nrf1 and NF-κB may be more complex than previously understood and warrants further exploration. Furthermore, acetylation promotes Nrf2 stabilization and nuclear translocation, which in turn activates downstream gene transcription [58], and the lysine‒arginine mutation at the acetylation site has no effect on the stability of the Nrf2 protein but disrupts the DNA-binding activity of Nrf2 in a promoter-specific manner [59]. Acetylation has been reported to mediate Nrf2 function in ischaemic stroke, and the histone deacetylase inhibitor trichostatin A (TSA) increases neuronal cell viability and reduces infarct volume, which is related to reduced expression of the Nrf2 suppressor Keap1, increased Keap1/Nrf2 dissociation and Nrf2 nuclear translocation [60]. In retinal I/R injury, Nrf2 contributes to the neuroprotective effect of TSA, which promotes Nrf2 nuclear translocation and acetylation [61]. A previous study revealed that acetyltransferase p300/CBP mediates the acetylation of Nrf1 and that Nrf1 acetylation promotes its own transcriptional activation by enhancing its ability to bind to DNA [62]. Nrf1 acetylation is reportedly reduced in the pericontusional cortex and affects transcriptional activity in this region after TBI [24]. Investigating the underlying mechanisms of reduced acetylation of Nrf1 remains critical. In this study, we demonstrated that PTS enhanced the acetylation and stability of Nrf1, which facilitated its nuclear localization. Additionally, PTS decreased the activity and expression of HDAC3 following ischaemia. Conversely, ITSA-1 activated HDAC3, thereby suppressing the neuroprotection provided by PTS and its anti-inflammatory effects in OGD/R-induced microglia. Furthermore, the HDAC3 inhibitor RGFP966 exacerbated the PTS-mediated decreases in p65, iNOS, IL-1β and TNF-α. This effect was also associated with the modulation of Nrf1 acetylation. Furthermore, we demonstrated that the K105R and K139R mutations in Nrf1 inhibited the stability of the Nrf1 protein. These mutations also reversed the improvement observed with PTS in terms of p65 and iNOS expression, as well as IL-1β and TNF-α levels, in OGD/R-induced microglia. Thus, the improvement in neurological function and the inflammatory response mediated by PTS might be related to HDAC3-mediated Nrf2 acetylation. Several studies on PTS support our findings. Among the different HDACs, HDAC3 is considered a drug target for epigenetic modulation and is currently being explored as a potential therapeutic strategy for refs. [34, 63]. ITSA-1 is an activator of histone deacetylases (HDACs) and counteracts TSA-induced histone acetylation and transcriptional activation [64], and thus ITSA-1 is used in experiments to determine the role of HDAC3 in inflammatory signalling [65]. The HDAC3–p65–cGAS–STING pathway in microglia plays a crucial role in neuroinflammation and tissue injury induced by ischaemic stroke. This pathway represents a novel therapeutic target that could offer new treatment approaches [66]. Huang et al. reported that esketamine improved post-stroke anxiety by regulating the HDAC3/NF-κB/COX1-mediated inflammatory response in microglia after ischaemic stroke [67]. Unlike reports indicating that HDAC3 directly mediates p65, our findings suggest that Nrf1 acetylation may serve as a link between HDAC3 and p65 interaction in microglia following ischaemic stroke. HDAC3 deacetylates Nrf1 within the cell nucleus, thereby facilitating the interaction between Nrf1 and p65 and subsequent p65 accumulation, which contributes to the anti-inflammatory effects of PTS. However, the intermediate regulatory functions of Nrf1 may represent a more intricate regulatory mechanism that requires further investigation.

## Conclusion

We have demonstrated that PTS decreases HDAC3 expression and activity, increases Nrf1 acetylation in the cell nucleus, and subsequently inhibits the interaction between Nrf1 and p65, as well as p65 accumulation in microglia. This contributes to a reduction in infarct volume and neuroinflammation (iNOS/Arg1, TNF-α, and IL-1β levels) after ischaemic stroke, and Nrf1 acetylation plays a crucial role in this process. The HDAC3/Nrf1/p65 pathway was identified for the first time as a potential regulatory mechanism underlying PTS neuroprotection, which sheds new light on the potential translational applications of PTS in clinical settings.

### Supplementary Information

Supplementary Material 1.

## Data Availability

The data that support the findings of this study are available from the corresponding author, Chi Zhang, upon request.

## Abbreviations

BDNF: Brain-derived neurotrophic factor
CHX: Cycloheximide
CNS: Central nervous system
HDAC3: Histone deacetylases 3
iNOS: Inducible nitric oxide synthase
I/R: Ischemia/reperfusion
MCAO/R: Middle cerebral artery occlusion–reperfusion
Nrf1: Nuclear factor (erythroid-derived 2)-like 1
OGD/R: Oxygen–glucose deprivation/reperfusion
PTS: Pterostilbene
RNS: Reactive nitrogen species
ROS: Reactive oxygen species
TBI: Traumatic brain injury
TTC: 2,3,5-Triphenyl tetrazolium chloride
WT: Wide type

## References

[1] Virani SS, Alonso A, Aparicio HJ, Benjamin EJ, Bittencourt MS, Callaway CW, et al. Heart disease and stroke statistics-2021 update: a report from the American Heart Association. Circulation. 2021;143(8):e254–743. 33501848 10.1161/CIR.0000000000000950 PMC13036842

[2] GBD 2019 Stroke Collaborators. Global, regional, and national burden of stroke and its risk factors, 1990–2019: a systematic analysis for the Global Burden of Disease Study 2019. Lancet Neurol. 2021;20(10):795–820. 34487721 10.1016/S1474-4422(21)00252-0 PMC8443449

[3] Paul S, Candelario-Jalil E. Emerging neuroprotective strategies for the treatment of ischemic stroke: an overview of clinical and preclinical studies. Exp Neurol. 2021;335:113518. 33144066 10.1016/j.expneurol.2020.113518 PMC7869696

[4] Herpich F, Rincon F. Management of acute ischemic stroke. Crit Care Med. 2020;48(11):1654–63. 32947473 10.1097/CCM.0000000000004597 PMC7540624

[5] Wu S, Wu B, Liu M, Chen Z, Wang W, Anderson CS, et al. Stroke in China: advances and challenges in epidemiology, prevention, and management. Lancet Neurol. 2019;18(4):394–405. 30878104 10.1016/S1474-4422(18)30500-3

[6] Hu S, Cui B, Mlynash M, Zhang X, Mehta KM, Lansberg MG. Stroke epidemiology and stroke policies in China from 1980 to 2017: a systematic review and meta-analysis. Int J Stroke. 2020;15(1):18–28. 31543073 10.1177/1747493019873562

[7] Boese AC, Lee JP, Hamblin MH. Neurovascular protection by peroxisome proliferator-activated receptor alpha in ischemic stroke. Exp Neurol. 2020;331:113323. 32320699 10.1016/j.expneurol.2020.113323 PMC13082750

[8] Gogoleva VS, Drutskaya MS, Atretkhany KS. The role of microglia in the homeostasis of the central nervous system and neuroinflammation. Mol Biol. 2019;53(5):790–8. 10.1134/S0026893319050054 31661478

[9] Kwon HS, Koh SH. Neuroinflammation in neurodegenerative disorders: the roles of microglia and astrocytes. Transl Neurodegener. 2020;9(1):42. 33239064 10.1186/s40035-020-00221-2 PMC7689983

[10] Jayaraj RL, Azimullah S, Beiram R, Jalal FY, Rosenberg GA. Neuroinflammation: friend and foe for ischemic stroke. J Neuroinflamm. 2019;16(1):142. 10.1186/s12974-019-1516-2 PMC6617684 31291966

[11] Chen Y, Gong K, Guo L, Zhang B, Chen S, Li Z, et al. Downregulation of phosphoglycerate mutase 5 improves microglial inflammasome activation after traumatic brain injury. Cell Death Discov. 2021;7(1):290. 34642327 10.1038/s41420-021-00686-8 PMC8511105

[12] Chu H, Huang C, Zhou Z, Tang Y, Dong Q, Guo Q. Inflammatory score predicts early hematoma expansion and poor outcomes in patients with intracerebral hemorrhage. Int J Surg. 2023;109(3):266–76. 37093070 10.1097/JS9.0000000000000191 PMC10389560

[13] Chen Y, Long T, Chen J, Wei H, Meng J, Kang M, et al. WTAP participates in neuronal damage by protein translation of NLRP3 in an m6A-YTHDF1-dependent manner after traumatic brain injury. Int J Surg. 2024. 10.1097/JS9.0000000000001794. 38874470 10.1097/JS9.0000000000001794 PMC11392096

[14] Zhang S. Microglial activation after ischaemic stroke. Stroke Vasc Neurol. 2019;4(2):71–4. 31338213 10.1136/svn-2018-000196 PMC6613941

[15] Deng W, Mandeville E, Terasaki Y, Li W, Holder J, Chuang AT, et al. Transcriptomic characterization of microglia activation in a rat model of ischemic stroke. J Cereb Blood Flow Metab. 2020;40(suppl):34–48. 10.1177/0271678X20932870. 10.1177/0271678X20932870 PMC7687036 33208001

[16] Qin C, Zhou LQ, Ma XT, Hu ZW, Yang S, Chen M, et al. Dual functions of microglia in ischemic stroke. Neurosci Bull. 2019;35(5):921–33. 31062335 10.1007/s12264-019-00388-3 PMC6754485

[17] Jiang CT, Wu WF, Deng YH, Ge JW. Modulators of microglia activation and polarization in ischemic stroke (review). Mol Med Rep. 2020;21(5):2006–18. 32323760 10.3892/mmr.2020.11003 PMC7115206

[18] Amato S, Arnold A. Modeling microglia activation and inflammation-based neuroprotectant strategies during ischemic stroke. Bull Math Biol. 2021;83(6):72. 33982158 10.1007/s11538-021-00905-4

[19] Zhang M, Wu Q, Tang M, Chen Z, Wu H. Exosomal Mir-3613-3p derived from oxygen-glucose deprivation-treated brain microvascular endothelial cell promotes microglial M1 polarization. Cell Mol Biol Lett. 2023;28(1):18. 36870962 10.1186/s11658-023-00432-1 PMC9985860

[20] Wufuer R, Fan Z, Liu K, Zhang Y. Differential yet integral contributions of Nrf1 and Nrf2 in the human HepG2 cells on antioxidant cytoprotective response against tert-butylhydroquinone as a pro-oxidative stressor. Antioxidants. 2021;10(10):1610. 34679746 10.3390/antiox10101610 PMC8533631

[21] Hu S, Feng J, Wang M, Wufuer R, Liu K, Zhang Z, et al. Nrf1 is an indispensable redox-determining factor for mitochondrial homeostasis by integrating multi-hierarchical regulatory networks. Redox Biol. 2022;57:102470. 36174386 10.1016/j.redox.2022.102470 PMC9520269

[22] Wang X, Chen G, Wan B, Dong Z, Xue Y, Luo Q, et al. NRF1-mediated microglial activation triggers high-altitude cerebral edema. J Mol Cell Biol. 2022;14(5):036. 10.1093/jmcb/mjac036. 10.1093/jmcb/mjac036 PMC9486928 35704676

[23] Wang X, Huang L, Jiang S, Cheng K, Wang D, Luo Q, et al. Testosterone attenuates pulmonary epithelial inflammation in male rats of COPD model through preventing NRF1-derived NF-κB signaling. J Mol Cell Biol. 2021;13(2):128–40. 33475136 10.1093/jmcb/mjaa079 PMC8104951

[24] Saha P, Gupta R, Sen T, Sen N. Activation of cyclin D1 affects mitochondrial mass following traumatic brain injury. Neurobiol Dis. 2018;118:108–16. 30010002 10.1016/j.nbd.2018.07.010 PMC6087674

[25] Fan H, Ding R, Liu W, Zhang X, Li R, Wei B, et al. Heat shock protein 22 modulates NRF1/TFAM-dependent mitochondrial biogenesis and DRP1-sparked mitochondrial apoptosis through AMPK-PGC1α signaling pathway to alleviate the early brain injury of subarachnoid hemorrhage in rats. Redox Biol. 2021;40:101856. 33472123 10.1016/j.redox.2021.101856 PMC7816003

[26] Yang M, Shen Y, Zhao S, Zhang R, Dong W, Lei X. Protective effect of resveratrol on mitochondrial biogenesis during hyperoxia-induced brain injury in neonatal pups. BMC Neurosci. 2023;24(1):27. 37098490 10.1186/s12868-023-00797-1 PMC10127954

[27] Yin W, Signore AP, Iwai M, Cao G, Gao Y, Chen J. Rapidly increased neuronal mitochondrial biogenesis after hypoxic-ischemic brain injury. Stroke. 2008;39(11):3057–63. 18723421 10.1161/STROKEAHA.108.520114 PMC2726706

[28] Hseu YC, Vudhya Gowrisankar Y, Wang LW, Zhang YZ, Chen XZ, Huang PJ, et al. The in vitro and in vivo depigmenting activity of pterostilbene through induction of autophagy in melanocytes and inhibition of UVA-irradiated α-MSH in keratinocytes via Nrf2-mediated antioxidant pathways. Redox Biol. 2021;44:102007. 34049220 10.1016/j.redox.2021.102007 PMC8167190

[29] Lin WS, Leland JV, Ho CT, Pan MH. Occurrence, bioavailability, anti-inflammatory, and anticancer effects of pterostilbene. J Agric Food Chem. 2020;68(46):12788–99. 32064876 10.1021/acs.jafc.9b07860

[30] Meng J, Chen Y, Bi F, Li H, Chang C, Liu W. Pterostilbene attenuates amyloid-β induced neurotoxicity with regulating PDE4A-CREB-BDNF pathway. Am J Transl Res. 2019;11(10):6356–69. 31737188 PMC6834512

[31] Liu J, He J, Huang Y, Hu Z. Resveratrol has an overall neuroprotective role in ischemic stroke: a meta-analysis in rodents. Front Pharmacol. 2021;12:795409. 34987407 10.3389/fphar.2021.795409 PMC8721173

[32] Nirwane A, Majumdar A. Resveratrol and pterostilbene attenuated smokeless tobacco induced cardiovascular aberrations in estrogen deficient female rats. Toxicol Res. 2016;5(6):1604–18. 10.1039/C6TX00225K PMC6062250 30090461

[33] Liu H, Wu X, Luo J, Wang X, Guo H, Feng D, et al. Pterostilbene attenuates astrocytic inflammation and neuronal oxidative injury after ischemia-reperfusion by inhibiting nf-κb phosphorylation. Front Immunol. 2019;10:2408. 31681297 10.3389/fimmu.2019.02408 PMC6811521

[34] Lu H, Ashiqueali R, Lin CI, Walchale A, Clendaniel V, Matheson R, et al. Histone deacetylase 3 inhibition decreases cerebral edema and protects the blood–brain barrier after stroke. Mol Neurobiol. 2023;60(1):235–46. 36258136 10.1007/s12035-022-03083-z PMC9758108

[35] Cai L, Gong Q, Qi L, Xu T, Suo Q, Li X, et al. ACT001 attenuates microglia-mediated neuroinflammation after traumatic brain injury via inhibiting AKT/NFκB/NLRP3 pathway. Cell Commun Signal. 2022;20(1):56. 35461293 10.1186/s12964-022-00862-y PMC9035258

[36] Chen Y, He W, Wei H, Chang C, Yang L, Meng J, et al. Srs11-92, a ferrostatin-1 analog, improves oxidative stress and neuroinflammation via Nrf2 signal following cerebral ischemia/reperfusion injury. CNS Neurosci Ther. 2023;29(6):1667–77. 36852441 10.1111/cns.14130 PMC10173707

[37] Chen Z, Wang X, Wu H, Fan Y, Yan Z, Lu C, et al. box binding protein 1 as a key modulator in “healing endothelial cells”, a novel EC phenotype promoting angiogenesis after MCAO. Cell Mol Biol Lett. 2022;27(1):97. 36348288 10.1186/s11658-022-00399-5 PMC9644469

[38] Zou X, Wang L, Wang S, Zhang Y, Ma J, Chen L, et al. Promising therapeutic targets for ischemic stroke identified from plasma and cerebrospinal fluid proteomes: a multicenter Mendelian randomization study. Int J Surg. 2024;110(2):766–76. 38016292 10.1097/JS9.0000000000000922 PMC10871597

[39] Yu X, Liu R, Gao W, Wang X, Zhang Y. Single-cell omics traces the heterogeneity of prostate cancer cells and the tumor microenvironment. Cell Mol Biol Lett. 2023;28(1):38. 37161356 10.1186/s11658-023-00450-z PMC10170780

[40] Hamner MA, McDonough A, Gong DC, Todd LJ, Rojas G, Hodecker S, et al. Microglial depletion abolishes ischemic preconditioning in white matter. Glia. 2022;70(4):661–74. 34939240 10.1002/glia.24132 PMC8994687

[41] Gharavi AT, Hanjani NA, Movahed E, Doroudian M. The role of macrophage subtypes and exosomes in immunomodulation. Cell Mol Biol Lett. 2022;27(1):83. 36192691 10.1186/s11658-022-00384-y PMC9528143

[42] Ren H, Pan Y, Wang D, Hao H, Han R, Qi C, et al. CD22 blockade modulates microglia activity to suppress neuroinflammation following intracerebral hemorrhage. Pharmacol Res. 2023;196:106912. 37696483 10.1016/j.phrs.2023.106912

[43] Liu J, Xu J, Mi Y, Yang Y, Li Q, Zhou D, et al. Pterostilbene alleviates cerebral ischemia and reperfusion injury in rats by modulating microglial activation. Food Funct. 2020;11(6):5432–45. 32490497 10.1039/D0FO00084A

[44] Maida CD, Norrito RL, Daidone M, Tuttolomondo A, Pinto A. Neuroinflammatory mechanisms in ischemic stroke: focus on cardioembolic stroke, background, and therapeutic approaches. Int J Mol Sci. 2020;21(18):6454. 32899616 10.3390/ijms21186454 PMC7555650

[45] Martha SR, Cheng Q, Fraser JF, Gong L, Collier LA, Davis SM, et al. Expression of cytokines and chemokines as predictors of stroke outcomes in acute ischemic stroke. Front Neurol. 2020;10:1391. 32010048 10.3389/fneur.2019.01391 PMC6974670

[46] Wytrykowska A, Prosba-Mackiewicz M, Nyka WM. IL-1beta, TNF-alpha, and IL-6 levels in gingival fluid and serum of patients with ischemic stroke. J Oral Sci. 2016;58(4):509–13. 28025434 10.2334/josnusd.16-0278

[47] Zhao SC, Ma LS, Chu ZH, Xu H, Wu WQ, Liu F. Regulation of microglial activation in stroke. Acta Pharmacol Sin. 2017;38(4):445–58. 28260801 10.1038/aps.2016.162 PMC5386316

[48] Nagy EE, Frigy A, Szász JA, Horváth E. Neuroinflammation and microglia/macrophage phenotype modulate the molecular background of post-stroke depression: a literature review. Exp Ther Med. 2020;20(3):2510–23. 32765743 10.3892/etm.2020.8933 PMC7401670

[49] Kawalec M, Wojtyniak P, Bielska E, Lewczuk A, Boratyńska-Jasińska A, Beręsewicz-Haller M, et al. Mitochondrial dynamics, elimination and biogenesis during post-ischemic recovery in ischemia-resistant and ischemia-vulnerable gerbil hippocampal regions. Biochim Biophys Acta Mol Basis Dis. 2023;1869(3):166633. 36566873 10.1016/j.bbadis.2022.166633

[50] Hu G, Zhou C, Wang J, Ma X, Ma H, Yu H, et al. Electroacupuncture treatment ameliorates depressive-like behavior and cognitive dysfunction via CB1R dependent mitochondria biogenesis after experimental global cerebral ischemic stroke. Front Cell Neurosci. 2023;17:1135227. 37091920 10.3389/fncel.2023.1135227 PMC10113634

[51] Bartelt A, Widenmaier SB, Schlein C, Johann K, Goncalves RLS, Eguchi K, et al. Brown adipose tissue thermogenic adaptation requires Nrf1-mediated proteasomal activity. Nat Med. 2018;24(3):292–303. 29400713 10.1038/nm.4481 PMC5839993

[52] Wang H, Zhu J, Liu Z, Lv H, Lv P, Chen F, et al. Silencing of long isoforms of nuclear factor erythroid 2 like 1 primes macrophages towards M1 polarization. Free Radic Biol Med. 2018;117:37–44. 29421237 10.1016/j.freeradbiomed.2018.01.022

[53] Ma L, Niu W, Lv J, Jia J, Zhu M, Yang S. PGC-1α-mediated mitochondrial biogenesis is involved in cannabinoid receptor 2 agonist AM1241-induced microglial phenotype amelioration. Cell Mol Neurobiol. 2018;38(8):1529–37. 30315387 10.1007/s10571-018-0628-z PMC11469869

[54] Pradeepkiran JA, Hindle A, Kshirsagar S, Reddy PH. Are mitophagy enhancers therapeutic targets for Alzheimer’s disease? Biomed Pharmacother. 2022;149:112918. 35585708 10.1016/j.biopha.2022.112918 PMC9148418

[55] Cheng K, Zhu L, Wang XT. Nuclear respiratory factor 1 mediates LPS-induced acute lung injury through NF-κB. Sheng Li Xue Bao. 2022;74(3):401–10. 35770638

[56] Suliman HB, Sweeney TE, Withers CM, Piantadosi CA. Co-regulation of nuclear respiratory factor-1 by NFkappaB and CREB links LPS-induced inflammation to mitochondrial biogenesis. J Cell Sci. 2010;123(Pt 15):2565–75. 20587593 10.1242/jcs.064089 PMC2912462

[57] Liang Y, Liu W, Zhao M, Shi D, Zhang Y, Luo B. Nuclear respiratory factor 1 promotes the progression of EBV-associated gastric cancer and maintains EBV latent infection. Virus Genes. 2023;59(2):204–14. 36738378 10.1007/s11262-023-01970-8

[58] Kawai Y, Garduño L, Theodore M, Yang J, Arinze IJ. Acetylation-deacetylation of the transcription factor Nrf2 (nuclear factor erythroid 2-related factor 2) regulates its transcriptional activity and nucleocytoplasmic localization. J Biol Chem. 2011;286(9):7629–40. 21196497 10.1074/jbc.M110.208173 PMC3045017

[59] Sun Z, Chin YE, Zhang DD. Acetylation of Nrf2 by p300/CBP augments promoter-specific DNA binding of Nrf2 during the antioxidant response. Mol Cell Biol. 2009;29(10):2658–72. 19273602 10.1128/MCB.01639-08 PMC2682049

[60] Wang B, Zhu X, Kim Y, Li J, Huang S, Saleem S, et al. Histone deacetylase inhibition activates transcription factor Nrf2 and protects against cerebral ischemic damage. Free Radic Biol Med. 2012;52(5):928–36. 22226832 10.1016/j.freeradbiomed.2011.12.006 PMC6010182

[61] Zhou C, Luo D, Xia W, Gu C, Lahm T, Xu X, et al. Nuclear factor (erythroid-derived 2)-like 2 (Nrf2) contributes to the neuroprotective effects of histone deacetylase inhibitors in retinal ischemia-reperfusion injury. Neuroscience. 2019;418:25–36. 31442569 10.1016/j.neuroscience.2019.08.027

[62] Izumi H, Ohta R, Nagatani G, Ise T, Nakayama Y, Nomoto M, et al. p300/CBP-associated factor (P/CAF) interacts with nuclear respiratory factor-1 to regulate the UDP-N-acetyl-alpha-d-galactosamine: polypeptide N-acetylgalactosaminyltransferase-3 gene. Biochem J. 2003;373(Pt 3):713–22. 12720548 10.1042/bj20021902 PMC1223531

[63] Zhou X, Qiao B. Inhibition of HDAC3 and ATXN3 by miR-25 prevents neuronal loss and ameliorates neurological recovery in cerebral stroke experimental rats. J Physiol Biochem. 2022;78(1):139–49. 35025075 10.1007/s13105-021-00848-3

[64] Koeller KM, Haggarty SJ, Perkins BD, Leykin I, Wong JC, Kao MC, et al. Chemical genetic modifier screens: small molecule trichostatin suppressors as probes of intracellular histone and tubulin acetylation. Chem Biol. 2003;10(5):397–410. 12770822 10.1016/S1074-5521(03)00093-0

[65] Behera J, Kelly KE, Voor MJ, Metreveli N, Tyagi SC, Tyagi N. Hydrogen sulfide promotes bone homeostasis by balancing inflammatory cytokine signaling in CBS-deficient mice through an epigenetic mechanism. Sci Rep. 2018;8(1):15226. 30323246 10.1038/s41598-018-33149-9 PMC6189133

[66] Liao Y, Cheng J, Kong X, Li S, Li X, Zhang M, et al. HDAC3 inhibition ameliorates ischemia/reperfusion-induced brain injury by regulating the microglial cGAS-STING pathway. Theranostics. 2020;10(21):9644–62. 32863951 10.7150/thno.47651 PMC7449914

[67] Huang A, Chen Y, Wang S, Du H, Guan A, Wu H, et al. Esketamine ameliorates post-stroke anxiety by modulating microglial HDAC3/NF-κB/COX1 inflammatory signaling in ischemic cortex. Eur J Pharmacol. 2023;947:175667. 36997050 10.1016/j.ejphar.2023.175667

