# Supplementary figures and images for: Pterostilbene improves neurological dysfunction and neuroinflammation after ischaemic stroke via HDAC3/Nrf1-mediated microglial activation
Source: Cell Mol Biol Lett. 2024 Aug 28;29:114. doi: 10.1186/s11658-024-00634-1 (PMC11360871; doi:10.1186/s11658-024-00634-1)

**Figure S1 The full uncropped Gels and Blots images.**

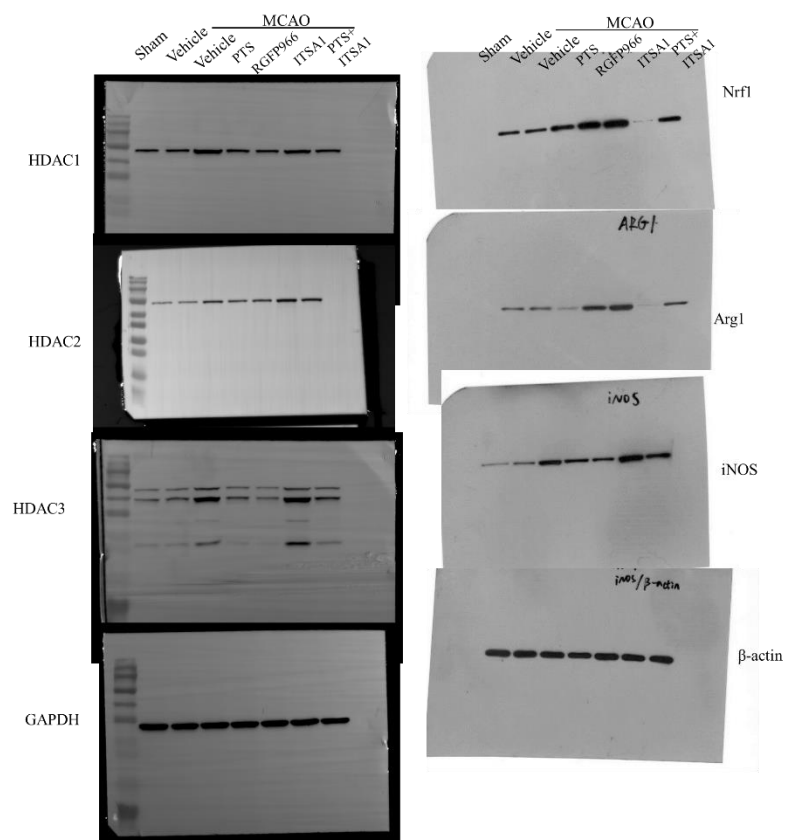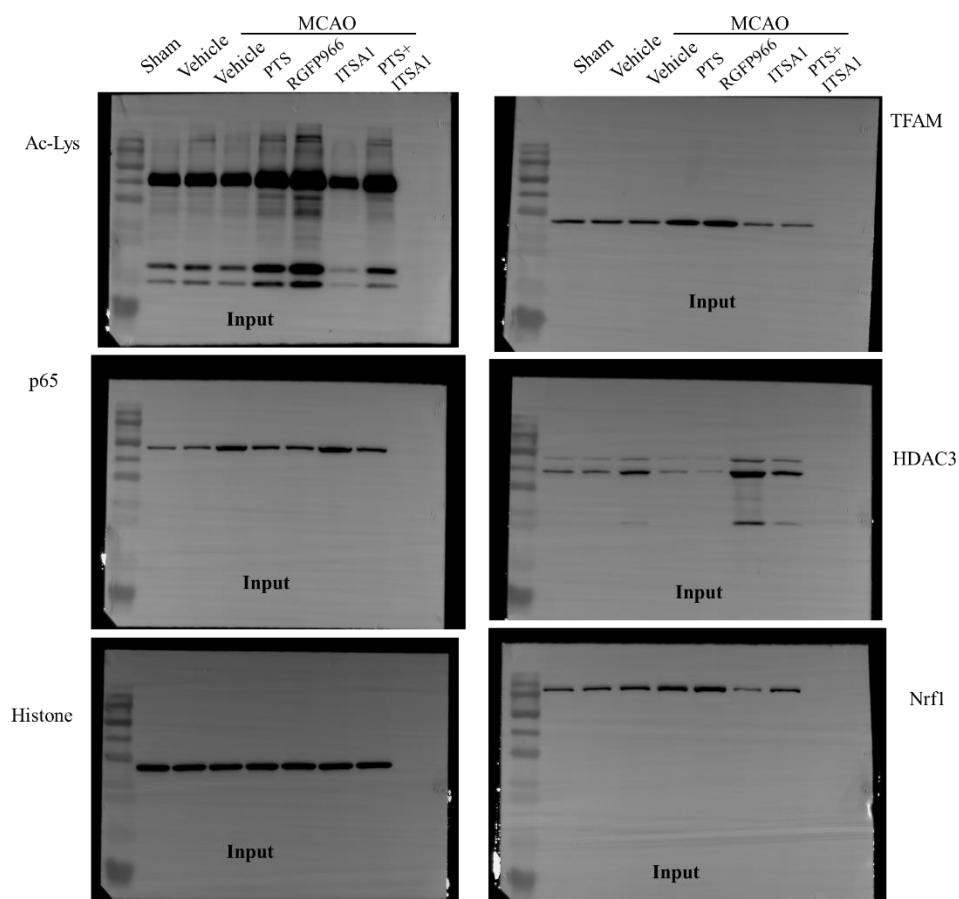

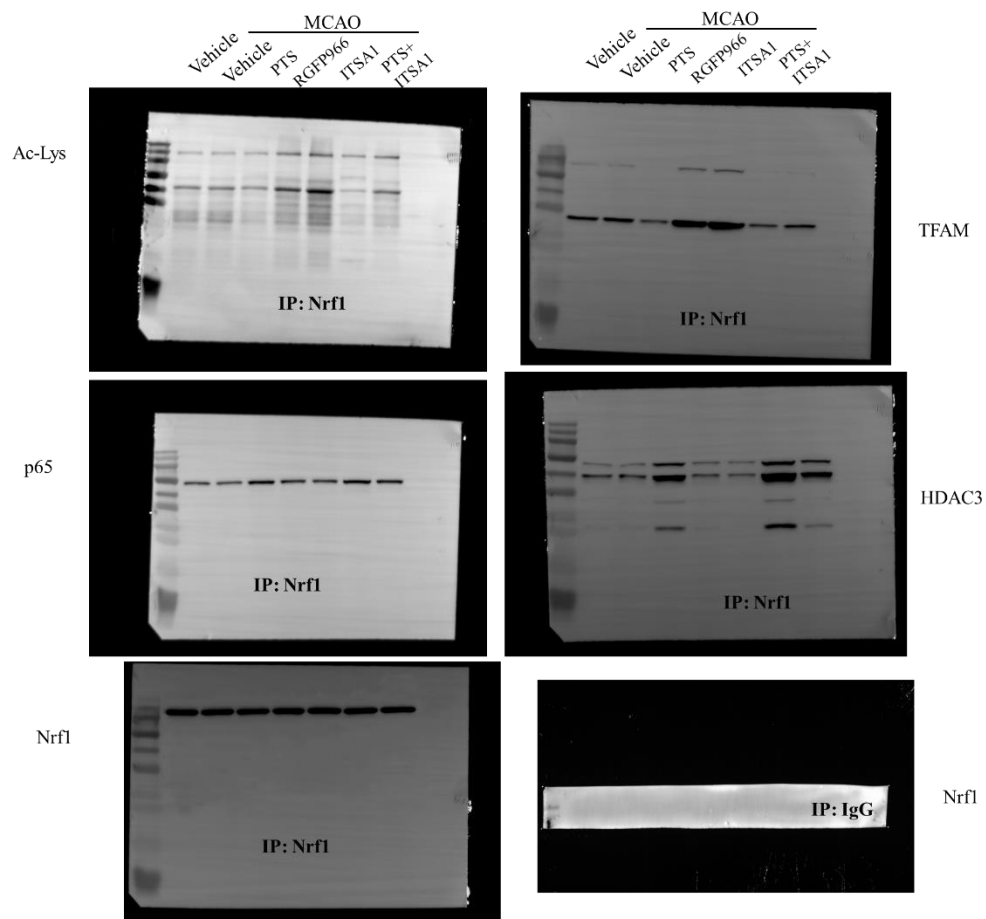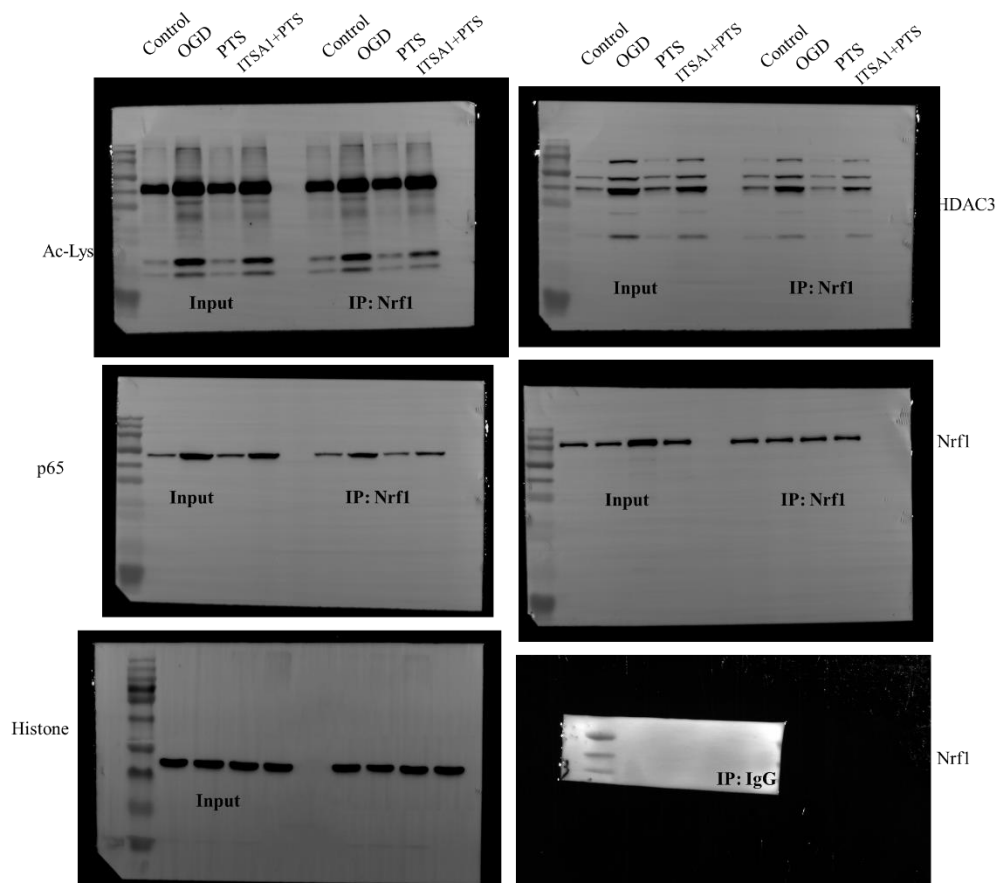

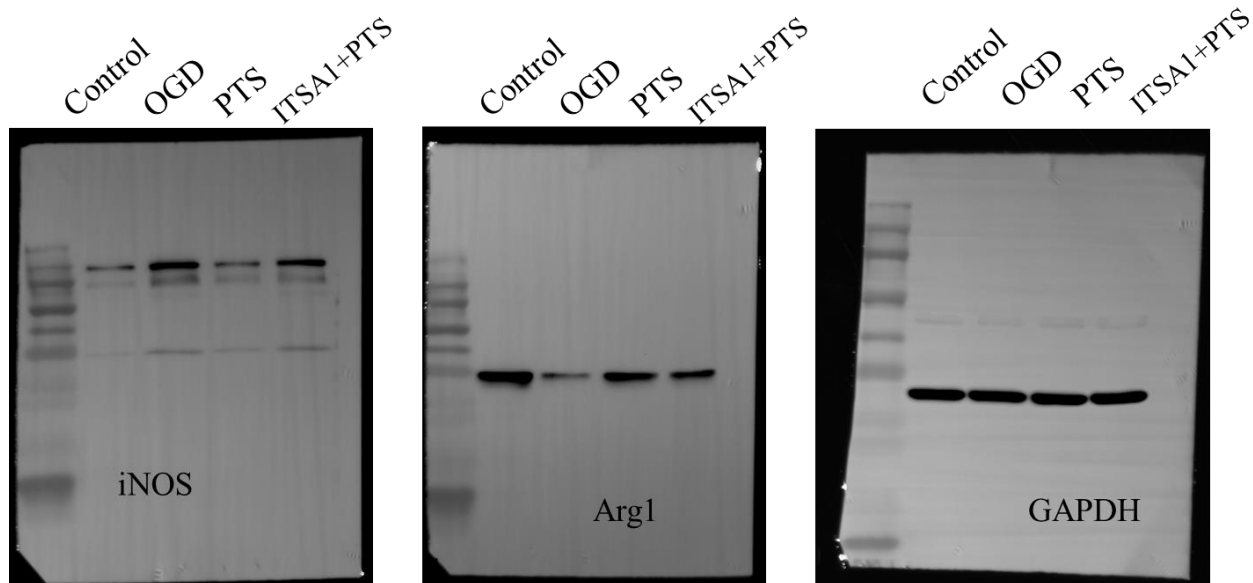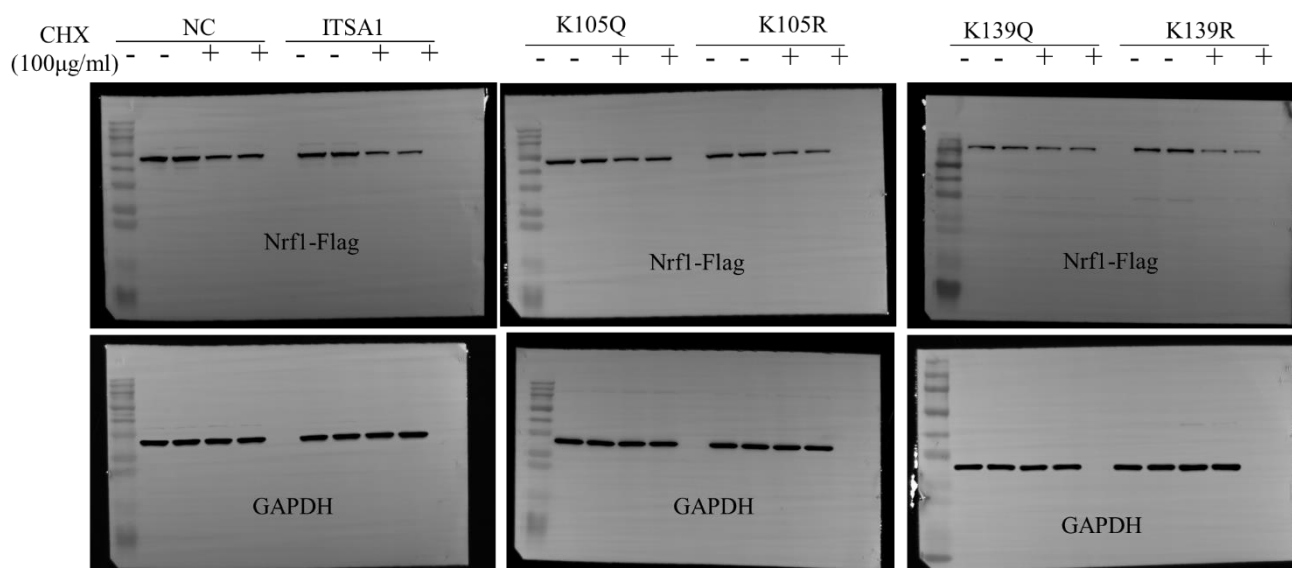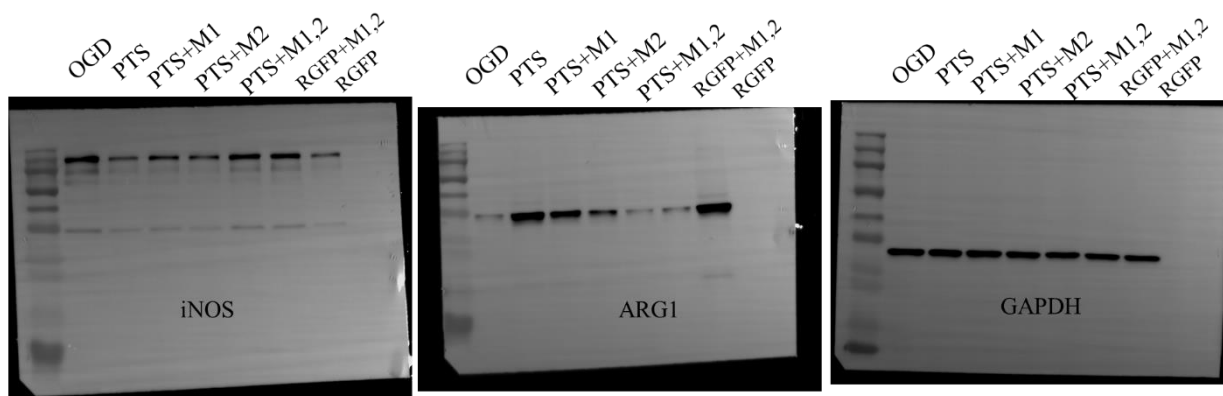

Supplement: Supplementary file 1 — Supplementary Material 1. [file 11658_2024_634_MOESM1_ESM.pdf]
